# The PDGF-BB-SOX7 axis-modulated IL-33 in pericytes and stromal cells promotes metastasis through tumour-associated macrophages

**DOI:** 10.1038/ncomms11385

**Published:** 2016-05-06

**Authors:** Yunlong Yang, Patrik Andersson, Kayoko Hosaka, Yin Zhang, Renhai Cao, Hideki Iwamoto, Xiaojuan Yang, Masaki Nakamura, Jian Wang, Rujie Zhuang, Hiromasa Morikawa, Yuan Xue, Harald Braun, Rudi Beyaert, Nilesh Samani, Susumu Nakae, Emily Hams, Steen Dissing, Padraic G. Fallon, Robert Langer, Yihai Cao

**Affiliations:** 1 Department of Microbiology, Tumor and Cell Biology, Karolinska Institute, 171 77 Stockholm, Sweden; 2 The TCM Hospital of Zhejiang Province, Hangzhou, Zhejiang 310006, China; 3 Unit of Computational Medicine, Department of Medicine, Center for Molecular Medicine, Karolinska Institute, 171 77 Stockholm, Sweden; 4 Institute for Medical Engineering and Science, Massachusetts Institute of Technology, Cambridge, Massachusetts 02139, USA; 5 Department of Biomedical Molecular Biology, Ghent University, B-9052 Ghent, Belgium; 6 Unit of Molecular Signal Transduction in Inflammation, Inflammation Research Center VIB, B-9052 Ghent, Belgium; 7 Department of Cardiovascular Sciences, University of Leicester and NIHR Leicester Cardiovascular Biomedical Research Unit, Glenfield Hospital, Leicester LE3 9QP, UK; 8 Laboratory of Systems Biology, Center for Experimental Medicine and Systems Biology, The Institute of Medical Science, The University of Tokyo, Tokyo 108–8639, Japan; 9 School of Medicine, Trinity Biomedical Sciences Institute, Trinity College Dublin, College Green, Dublin 2, Ireland; 10 Department of Cellular and Molecular Medicine, Panum Institute, University of Copenhagen, 2200N Copenhagen, Denmark; 11 Department of Medicine and Health Sciences, Linköping University, 581 83 Linköping, Sweden

## Abstract

Signalling molecules and pathways that mediate crosstalk between various tumour cellular compartments in cancer metastasis remain largely unknown. We report a mechanism of the interaction between perivascular cells and tumour-associated macrophages (TAMs) in promoting metastasis through the IL-33–ST2-dependent pathway in xenograft mouse models of cancer. IL-33 is the highest upregulated gene through activation of SOX7 transcription factor in PDGF-BB-stimulated pericytes. Gain- and loss-of-function experiments validate that IL-33 promotes metastasis through recruitment of TAMs. Pharmacological inhibition of the IL-33–ST2 signalling by a soluble ST2 significantly inhibits TAMs and metastasis. Genetic deletion of host IL-33 in mice also blocks PDGF-BB-induced TAM recruitment and metastasis. These findings shed light on the role of tumour stroma in promoting metastasis and have therapeutic implications for cancer therapy.

## Abstract (web summary)

Elevated IL-33 levels have been correlated with metastasis and poor prognosis. Here the authors show in mouse tumour xenograft models that PDGF-BB produced by tumour cells induces IL-33 via Sox7 in tumour pericytes, and IL-33 promotes metastasis through its effects on tumour-associated macrophages.

## Graphical abstract

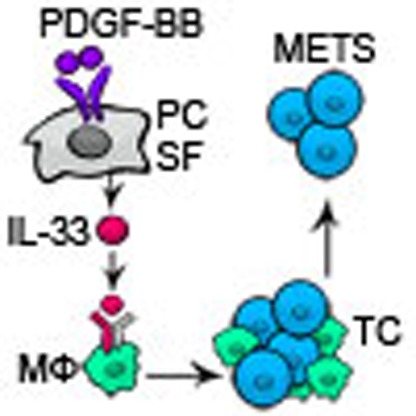

Cancer metastasis is a complex process that involves in sophisticated interactions between malignant and host cells^1^,^2^. Cancer cells often produce signalling molecules to manipulate host cells in the local microenvironment to facilitate their invasion, dissemination and metastasis. The PDGF-PDGFR signalling often becomes activated in the tumour microenvironment^3^,^4^,^5^ and endothelial cells in angiogenic vessels are an important source for the production of PDGF-BB^6^, a pluripotent member in the PDGF family. In epithelial cell- and other cell-originated cancer types, PDGF-BB primarily targets stromal fibroblasts and perivascular cells including pericytes and vascular smooth muscle cells^7^. PDGF-BB stimulates the proliferation and migration of perivascular cells through activation of PDGFRβ although interaction with PDGFRα also occurs in fibroblasts^5^,^7^. Although it is well known that PDGF-BB modulates vascular remodelling and maturation by recruiting pericytes and vascular smooth muscle cells onto angiogenic vessels, activation of these perivascular cells in the tumour microenvironment in cancer invasion and metastasis is poorly understood.

Tumour tissues often contain an exceptionally high number of inflammatory cells, which significantly alter tumour growth, angiogenesis, metastasis and drug responses^8^,^9^. Inflammatory cytokines including GM-CSF, TNF-α, IL-1β, IL-6 and various chemokines are actively involved in recruitment of inflammatory cells in tumours^10^,^11^. However, our current understanding of recruitment of tumour-associated macrophages (TAMs) and their roles in cancer invasion and metastasis are far from complete. IL-33 as a relatively new cytokine belongs to IL-1 family and it can be produced by a broad range of cell types including fibroblasts, osteoblasts, endothelial cells, epithelial cells and adipocytes^12^,^13^,^14^,^15^. IL-33 exerts its biological functions through binding and activation of its receptor ST2, a member in the Toll-like receptor superfamily. IL-33 is known to regulate Th2 immune responses^12^. However, the role of IL-33 in tumour inflammation and metastasis is unknown. A recent study shows that in a mouse breast cancer model, injection of IL-33 protein stimulates primary tumour growth and metastasis^16^.

In the present study, we show that IL-33 is the most upregulated gene in PDGF-BB-stimulated pericytes and SOX7 transcription factor mediates PDGF-BB-induced IL-33 expression. Gain-of-function and loss-of-function experiments demonstrate that pericyte- and stromal cell-derived IL-33 is a crucial cytokine for recruitment of TAMs in the tumour microenvironment. Importantly, in several human and mouse graft tumour models, we provide compelling evidence to demonstrate that pericyte- and stromal cell-derived IL-33-activated TAMs are crucial for cancer metastasis. Finally, in *in vivo* tumour models, we show that IL-33-activated TAMs mediate PDGF-BB-induced cancer metastasis. These findings shed new mechanistic lights on the crosstalk between various host cellular compartments and PDGF-BB-stimulated pericytes in promoting cancer metastasis. Functional blocking of the PDGF-BB-IL-33-TAM axis is an important approach for cancer therapy.

## Results

### PDGF-BB-PDGFRβ signalling indirectly recruits TAMs

**Figure 1:**
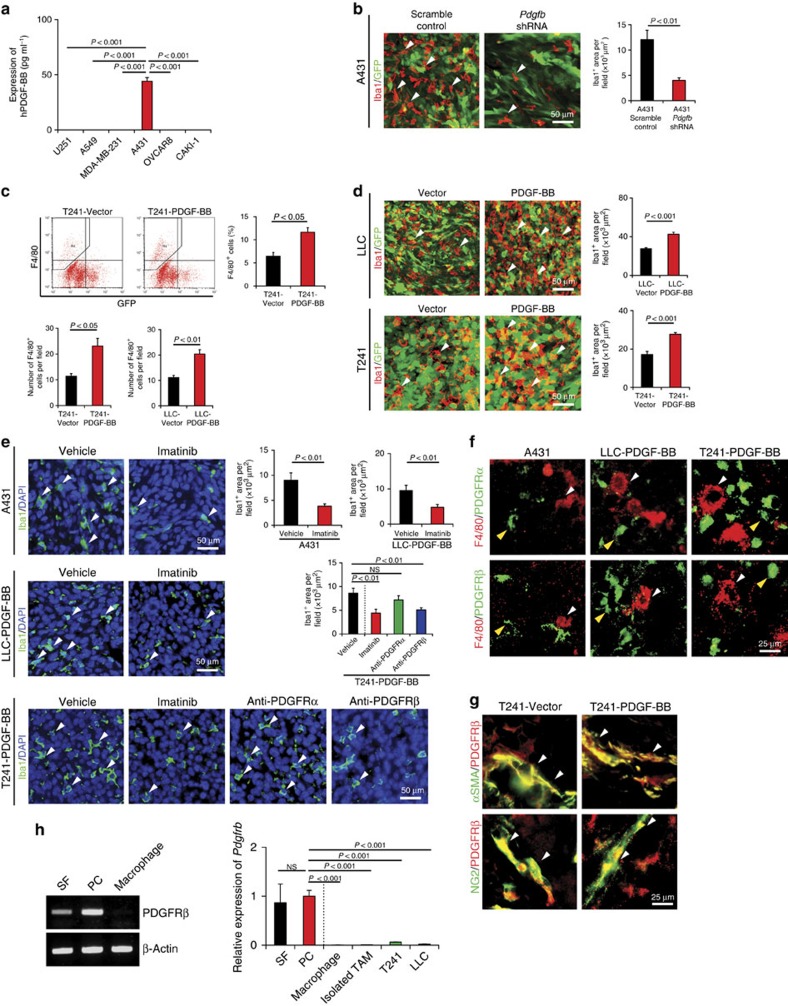
PDGF-BB induces PDGFRβ-dependent macrophage recruitment in tumour cell line grafts tumours of human and mouse origin. (**a**) Expression levels of PDGF-BB in conditioned medium of various human tumour cell lines (*n*=3 samples per group). (**b**) Iba1^+^ macrophages (red) in human scrambled shRNA-A431 and *Pdgfb* shRNA-A431 squamous carcinomas cell line grafts. Arrowheads indicate tumour-infiltrating macrophages. Scale bar, 50 μm. Iba1^+^ TAMs were quantified as areas (*n*=8 random fields per group). (**c**) Upper panels: FACS analysis of F4/80^+^ TAMs in vector-T241 and PDGF-BB-T241 fibrosarcoma cell line grafts and quantification of percentages of F4/80^+^ macrophages (*n*=6 samples per group). Lower panels: quantification of F4/80^+^ TAMs of immunohistochemical micrographs (*n*=8 random fields per group). (**d**) Iba1^+^ macrophages (red) in vector-T241 and PDGF-BB-T241, and vector- and PDGF-BB-LLC tumours. The tumour cells express GFP (green). Arrowheads indicate tumour-infiltrated macrophages. Scale bar, 50 μm. Iba1^+^ TAMs were quantified as areas (*n*=8 random fields per group). (**e**) Iba1^+^ macrophages (green) in vehicle- or imatinib-treated A431 and PDGF-BB-LLC tumours, and in vehicle-, imatinib-, anti-PDGFRα- or anti-PDGFRβ-treated PDGF-BB-T241 tumours. Tissue sections were counter-stained with DAPI (blue). Arrowheads indicate tumour-infiltrated macrophages. Scale bar, 50 μm. Iba1^+^ TAMs were quantified from eight random fields per group. (**f**) PDGFRβ (green) and F4/80 (red), or PDGFRα (green) and F4/80 (red) double immunostaining of A431, PDGF-BB-T241 and PDGF-BB-LLC tumours. White arrowheads indicate F4/80^+^ macrophages and yellow arrowheads point to PDGFRα^+^ or PDGFRβ^+^ cells. Scale bar, 25 μm. (**g**) αSMA (green) and PDGFRβ (red), or NG2 (green) and PDGFRβ (red) double immunostaining of vector- and PDGF-BB-T241 tumours. Arrowheads indicate double-positive signals (yellow). Scale bar, 25 μm. (**h**) RT–PCR and qPCR analyses of *Pdgfrb* in various cell types. Beta-actin was used as a standard loading (mean±s.e.m., NS, not significant, Student's *t*-test).

To investigate the role of PDGF-BB in the recruitment of TAMs, we screened a panel of human tumour cell lines that spontaneously express PDGF-BB. We have found that human A431 squamous carcinoma cell line expressed a high level of endogenous PDGF-BB protein (50 pg ml^−1^) (Fig. 1a). The A431 xenograft tumour contained a high number of Iba1^+^ TAMs (Fig. 1b). Interestingly, downregulation of PDGF-BB by *Pdgfb*-specific shRNA, which effectively inhibited the *Pdgfb* mRNA level (Supplementary Fig. 1a), markedly ablated TAMs in tumour tissues (Fig. 1b), suggesting that PDGF-BB was primarily responsible for TAM recruitment in this human xenograft model. To further validate these findings, we performed gain-of-function experiments in which mouse Lewis lung carcinoma (LLC) and T241 fibrosarcoma were transfected with *Pdgfb*-retrovirus to stably express PDGF-BB (Supplementary Fig. 1b and c). ShRNA knockdown of *Pdgfb* significantly inhibited A431 tumour growth (Supplementary Fig. 1d), whereas PDGF-BB expression promoted tumour growth in T241 and LLC tumours (Supplementary Fig. 1e and f). Notably, FACS and immunohistochemical analyses showed that PDGF-BB-LLC and T241 tumours contained significantly higher numbers of F4/80^+^ and Iba1^+^ TAMs as compared with their respective vector-transfected tumours (Fig. 1c,d). Of note, Iba1 and F4/80 double immunostaining showed completely overlapping positive signals (Supplementary Fig. 1g), indicating that both markers detect the total macrophage population in tumour tissues. These findings demonstrate that PDGF-BB recruits TAMs in human and mouse cell line-derived graft tumour models.

To define PDGFRs that are responsible for TAM recruitment, we used various PDGFR inhibitors. Imatinib, a pan PDGFR tyrosine kinase inhibitor^17^, significantly inhibited TAM recruitment in A431, LLC and T241 tumours (Fig. 1e), suggesting that PDGFRs mediate PDGF-BB-induced TAM infiltration. To distinguish PDGFRα and PDGFRβ signalling in TAM recruitment, anti-mouse PDGFRα- and PDGFRβ-specific neutralizing antibodies (PDGFR blockades) were used for the treatment of PDGF-BB^+^ T241 tumours. Interestingly, PDGFRβ, but not PDGFRα, blockade, markedly inhibited PDGF-BB-induced TAM infiltration (Fig. 1e). These findings indicate that PDGFRβ is the receptor that mediates PDGF-BB-induced TAM recruitment.

We next investigated the direct versus indirect role of PDGF-BB in the recruitment of TAMs. Surprisingly, co-localization of PDGFRα and PDGFRβ in T241 tumours by their specific antibodies showed that TAMs lacked PDGFR expression (Fig. 1f), suggesting an indirect role of PDGF-BB in the recruitment of TAMs. Consistent with this notion, PDGFRβ was primarily localized in non-TAM cells including NG2^+^ pericytes and αSMA^+^ smooth muscle cells (SMCs)/myofibroblasts (Fig. 1g). These findings were further quantitatively validated by PCR with reverse transcription (RT–PCR), quantitative PCR (qPCR) and staining of various cell lines showing that stromal fibroblasts and pericytes expressed high levels of *Pdgfrb* mRNA, whereas mouse Raw macrophage-like cell line and isolated TAMs completely lacked *Pdgfrb* mRNA expression (Fig. 1h and Supplementary Fig. 1h). These findings further support our notion that PDGF-BB recruits TAMs in various tumour models through an indirect mechanism.

### PDGF-BB induces pericyte- and fibroblast-derived IL-33

**Figure 2:**
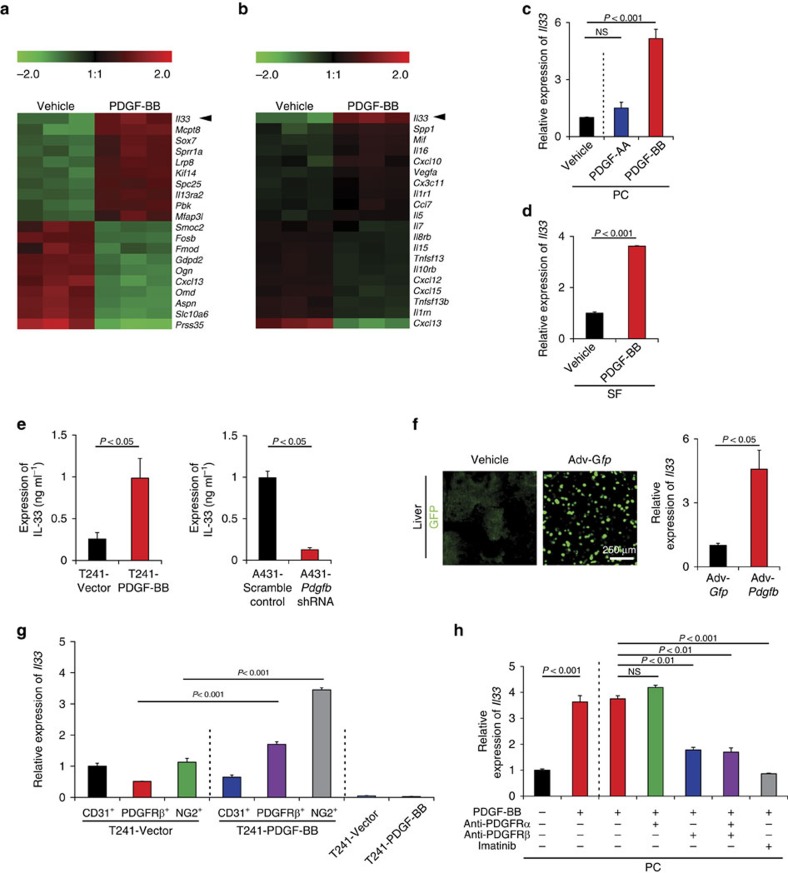
PDGF-BB-PDGFRβ-signalling induces IL-33 expression. (**a**) Heatmap of top 10 most upregulated and downregulated genes by genome-wide expression profiling of PDGF-BB-stimulated lung pericytes cultured *in vitro*. (**b**) Heatmap of top 10 most upregulated and downregulated inflammation-related signalling molecules by genome-wide expression profiling of PDGF-BB-stimulated lung pericytes cultured *in vitro*. (**c**) qPCR quantification of *Il33* mRNA expression levels in PDGF-AA- or PDGF-BB-stimulated lung pericytes cultured *in vitro*. (PC; *n*=6 samples per group). NS, not significant. (**d**) qPCR quantification of *Il33* mRNA expression levels in PDGF-BB-stimulated bone marrow stromal fibroblasts cultured *in vitro* (SF; *n*=6 samples per group). (**e**) Quantification of mouse IL-33 protein levels of vector- and PDGF-BB-T241 tumours, and scrambled and *Pdgfb* shRNA-transfected A431 tumours (*n*=6 samples per group). (**f**) qPCR quantification of *Il33* mRNA of Adv-*Gfp*- and Adv-*Pdgfb*-infected liver tissues (*n*=6 samples per group). Adv-*Gfp*-infected hepatocytes were visualized by a fluorescent microscope. (**g**) qPCR quantification of *Il33* mRNA expression levels in CD31^+^, PDGFRβ^+^ and NG2^+^ cell populations isolated from vector and PDGF-BB T241 tumours (*n*=6 samples per group). Vector and PDGF-BB T241 tumour cells served as controls. (**h**) qPCR analysis of *Il33* mRNA of vehicle-, anti-PDGFRα-, anti-PDGFRβ- or imatinib-treated PDGF-BB-stimulated or non-stimulated lung pericytes cultured *in vitro*. (PC; *n*=6 samples per group; mean±s.e.m., NS, not significant, Student's *t*-test).

To identify possible genes that mediate PDGF-BB-induced TAM recruitment, we performed a genome-wide expression microarray analysis in PDGF-BB-stimulated pericytes. Surprisingly, *Il33* was the most upregulated gene product with more than an eight-fold increase among all the genes in the genome (Fig. 2a), and was the top one of the upregulated inflammatory cytokines (Fig. 2b). The PDGF-BB-induced IL-33 expression was further validated by qPCR, which revealed more than a five-fold increase of *Il33* mRNA expression (Fig. 2c). In contrast, PDGF-AA, a ligand that only binds to PDGFRα, lacked ability to induce *Il33* expression (Fig. 2c), indicating that PDGFRβ is responsible for PDGF-BB-induced *Il33* expression. In addition to pericytes, stimulation of PDGFRβ^+^ stromal fibroblasts with PDGF-BB also led to marked upregulation of *Il33* mRNA (Fig. 2d). We further analysed the IL-33 protein expression from PDGF-BB-stimulated pericytes and stromal fibroblasts. Again, the IL-33 protein levels in PDGF-BB-stimulated pericytes and fibroblasts were significantly higher than those in non-stimulated cells (Supplementary Fig. 2a).

To validate these findings *in vivo*, we analysed IL-33 protein expression in PDGF-BB^+^ T241 tumours and found a marked increase of IL-33 expression as compared with vector tumours (Fig. 2e). IL-33 protein expression levels in A431 tumour grafts were markedly decreased by the *Pdgfb*-specific shRNA (Fig. 2e). We provided further *in vivo* evidence by delivery of adenoviral *Pdgfb* (Adv-*Pdgfb*) into tumour-free mice. Again, delivery of Adv-*Pdgfb* significantly induced IL-33 expression in the hepatic tissue (Fig. 2f). Collectively, these *in vitro* and *in vivo* findings provide compelling evidence that PDGF-BB markedly induces IL-33 expression in PDGFRβ^+^ perivascular cells and stromal fibroblasts.

To further validate the pericytes and stromal cells as the major source of IL-33 production in *in vivo* tumours, we isolated different cell types from the tumour microenvironment. We confirmed that the PDGFRβ^+^ cell population including stromal fibroblasts and perivascular cells were the important cells for the production of IL-33 in response to PDGF-BB (Fig. 2g). Furthermore, NG2^+^ pericytes in PDGF-BB tumours produced high levels of IL-33 as compared with those isolated from the vector control tumours (Fig. 2g). In contrast, CD31^+^ vascular endothelial cells did not significantly contribute to PDGF-BB-induced IL-33 expression in tumours since IL-33 levels in PDGF-BB positive population was not increased (Fig. 2g). Similarly, tumour cells produced negligible levels of IL-33 in PDGF-BB-positive and -negative tumour cells, which remained unchanged. Taken together, these findings demonstrate that pericytes and tumour stromal cells are the primary source of IL-33 in the tumour microenvironment.

We treated PDGF-BB-stimulated pericytes with PDGFRα and PDGFRβ blockades to monitor IL-33 expression *in vitro*. PDGFRβ, but not PDGFRα, specific blockade significantly inhibited PDGF-BB-induced IL-33 expression in pericytes (Fig. 2h). The combination of PDGFRβ and PDGFRα blockades did not produce any additive effects. Similar to PDGFRβ blockade, imatinib also produced a markedly inhibitory effect on IL-33 expression (Fig. 2h). Likewise, PDGFRβ blockade also significantly inhibited PDGF-BB-induced IL-33 expression in stromal fibroblasts (Supplementary Fig. 2b).

### Role of PDGF-BB signalling pathways in IL-33 production

**Figure 3:**
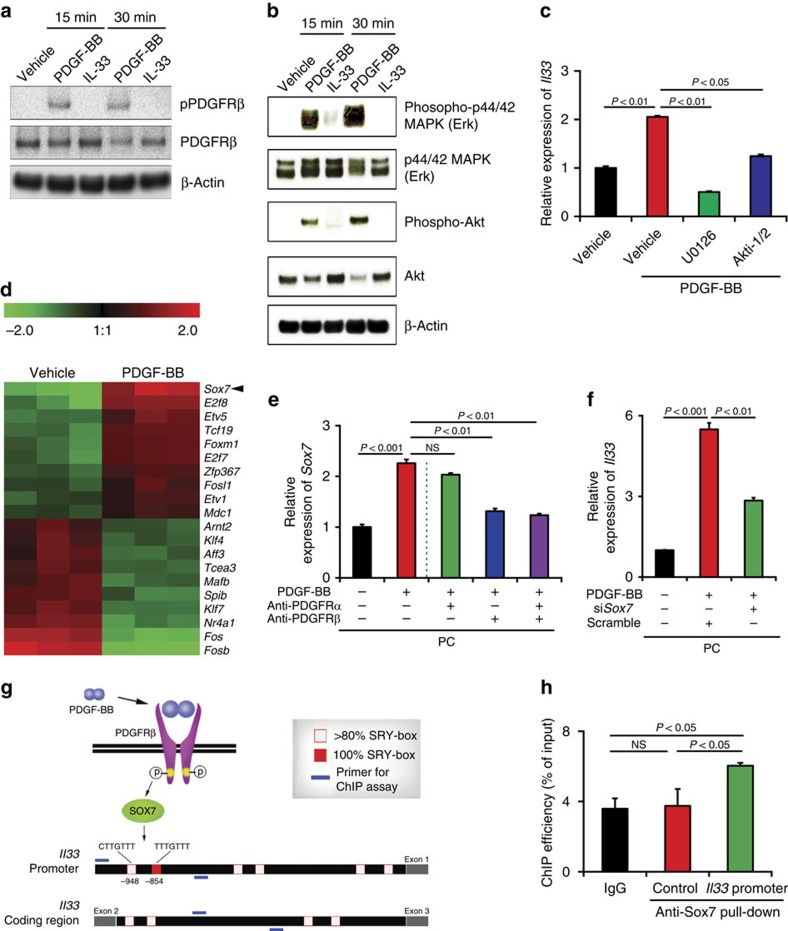
SOX7 transcription factor mediates the PDGF-BB-PDGFRβ-induced IL-33 expression. (**a**) Western immunoblotting analysis of phosphorylation of PDGFRβ of vehicle-, PDGF-BB- and IL-33-treated lung pericytes cultured *in vitro*. Beta-actin indicates loading levels. (**b**) Western immunoblotting analysis of Erk phosphorylation, Akt phosphorylation of vehicle-, PDGF-BB- and IL-33-treated pericytes. Beta-actin indicates loading levels. (**c**) qPCR analysis of *Il33* mRNA of vehicle-, U0126- or Akti-1/2-treated PDGF-BB-stimulated or non-stimulated lung pericytes cultured *in vitro*. (PC; *n*=6 samples per group). NS, not significant. (**d**) Heatmap profiling of transcription factor gene expression of vehicle- and PDGF-BB-stimulated lung pericytes cultured *in vitro*. (**e**) qPCR analysis of *Sox7* mRNA expression levels of anti-PDGFRα- or anti-PDGFRβ-treated lung pericytes that received PDGF-BB-stimulation (*n*=6 samples per group). Vehicle-treated pericytes served as controls (*n*=6 samples per group). NS, not significant; PC, pericyte. (**f**) qPCR analysis of *Il33* mRNA expression levels of vehicle- or PDGF-BB-stimulated lung pericytes that were transfected with scrambled or *Sox7* siRNA (*n*=6 samples per group). PC, pericyte. (**g**) Schematic diagram of IL-33 expression in pericytes regulated by the PDGF-BB-PDGFRβ signalling through SOX7 transcriptional regulation. PDGF-BB-activated PDGFRβ induces SOX7 that targets the SRY boxes located in the *Il33* promoter. (**h**) ChIP assay of SOX7 binding to the *Il33* gene promoter. Non-immune IgG and *Il33* coding region served as controls (*n*=6 samples per group) (mean±s.e.m., NS, not significant, Student's *t*-test). Full-gel images for **a**,**b** are shown in Supplementary Fig. 9.

Signalling pathway analysis showed that PDGF-BB induced activation of PDGFRβ by phosphorylation (Fig. 3a) and IL-33 has no impact on activation of PDGFRβ in pericytes. In concordance with the activation of PDGFRβ, downstream signalling components including MAP kinase (Erk) and Akt also became activated in PDGF-BB-stimulated pericytes (Fig. 3b). Signalling network analysis from cBioPortal^18^ showed that Akt and MAPK were correlated with PDGF-BB expression (Supplementary Fig. 3a). Consistently, MAPK and Akt-specific inhibitors significantly and effectively inhibited *Il33* mRNA expression levels in PDGF-BB-stimulated pericytes (Fig. 3c and Supplementary Fig. 3b). These findings show that PDGF-BB induces IL-33 expression in pericytes through activation of the PDGFRβ signalling pathway.

### SOX7 mediates PDGF-BB-induced IL-33 expression

We next investigated potential mechanisms by which PDGF-BB induces IL-33 expression in PDGFRβ^+^ pericytes and fibroblasts. Genome-wide microarray analysis of PDGF-BB-stimulated pericytes revealed that SOX7 was the most upregulated transcription factor (about six-fold; Fig. 3d), which was ranked as the top three most upregulated gene products in the genome (Fig. 2a and Supplementary Fig. 3c). The qPCR analysis further validated the increased expression level of *Sox7* mRNA in PDGF-BB-stimulated pericytes (Fig. 3e). Notably, PDGFRβ-specific blockade significantly attenuated PDGF-BB-stimulated expression of Sox7, whereas PDGFRα-specific blockade had no effect on *Sox7* mRNA expression (Fig. 3e). These findings suggest that PDGFRβ potentially mediates PDGF-BB-induced Sox7 expression.

To decipher the functional relation between SOX7 and IL-33 expression, PDGF-BB-stimulated pericytes were treated with *Sox7*-siRNA. Knockdown of SOX7 significantly impaired PDGF-BB-induced IL-33 expression (Fig. 3f), which was correlated to the knockdown efficiency (Supplementary Fig. 3d). Similarly, *Sox7*-siRNA knockdown also markedly reduced IL-33 production in PDGF-BB-stimulated stromal fibroblasts (Supplementary Fig. 3e). To provide further supportive evidence of transcriptional regulation of IL-33 expression by SOX7, we analysed mouse sequences of the IL-33 promoter region and discovered a canonical SOX7-binding SRY box and five non-canonical binding sites (Fig. 3g). Chromatin immunoprecipitation (ChIP) assay using the *Il33* promoter fragment containing the canonical binding site demonstrated that SOX7 directly bound to the *Il33* promoter (Fig. 3h). However, it is possible that the non-canonical SOX7 binding sites might also mediate direct binding of SOX7. These data show that PDGF-BB induces IL-33 expression through the PDGFRβ-SOX7 signalling pathway.

### Signalling mechanisms of IL-33-induced Raw cell migration

**Figure 4:**
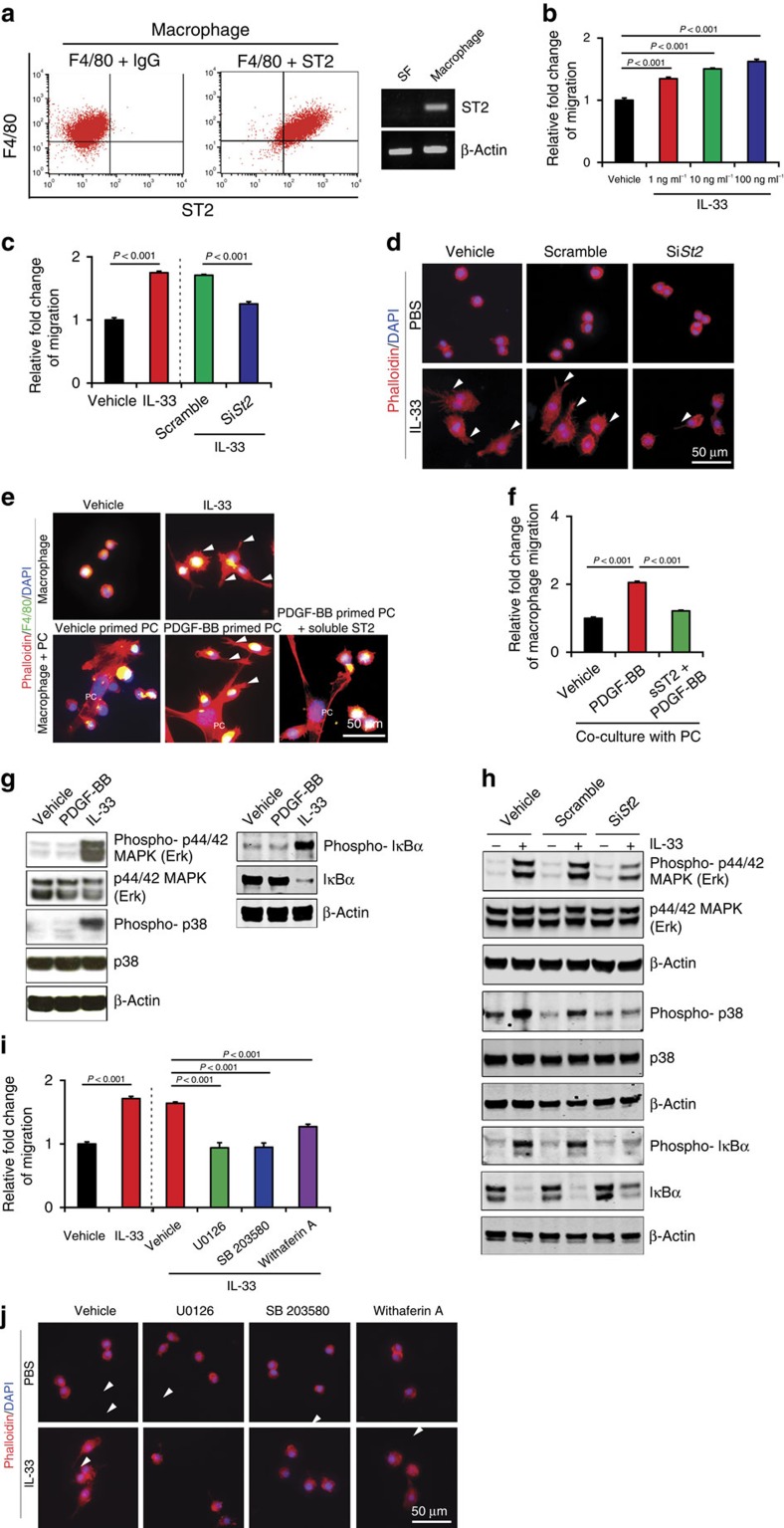
IL-33 stimulates Raw cell migration through activation of the ST2-intracellular signalling pathways. (**a**) Left panels: FACS analysis of F4/80^+^ST2^+^ mouse Raw macrophage cell line (*n*=6 samples per group). Non-immune IgG served as a negative control. Right panel: *St2* mRNA expression in macrophages and stromal fibroblasts. (**b**) Dose-dependent stimulation of Raw cell migration by IL-33 (*n*=6 samples per group). Vehicle-treated macrophages served as controls. (**c**) Inhibition of IL-33-induced Raw cell migration by a siRNA specifically targeting *St2* (*n*=6 samples per group). Scrambled siRNA serves as a control. (**d**) Inhibition of IL-33-induced morphological changes of Raw cells by a siRNA specifically targeting *St2*. Scrambled siRNA serves as a control. Arrowheads indicate filopodia sprouts of the IL-33-activated macrophages. Scale bar, 50 μm. (**e**) Cell morphologies of Raw cells co-cultured 48 h with PDGF-BB- or buffer-stimulated lung pericytes. F4/80 were shown in green (yellow overlapped with phalloidin red). Soluble ST2 was added to block IL-33 function (200 ng ml^−1^). PC, pericyte. Scale bar, 50 μm. Arrowheads indicate filopodia sprouts of the IL-33-activated macrophages. Recombinant IL-33-stimulated mouse Raw cells served as a positive control. (**f**) Migration of Raw cells co-cultured with PDGF-BB- or buffer-stimulated lung pericytes in the presence of a soluble ST2 or vehicle (*n*=6 samples per group). (**g**) IL-33 induces phosphorylation of MAPK and p38 at 10 min, and IκBα at 5 min in mouse Raw cells. Beta-actin indicates the loading level in each lane. (**h**) SiRNA specifically targeting *St2* inhibited IL-33-induced phosphorylation of MAPK, p38 and IκBα in Raw cells. Beta-actin indicates the loading level in each lane. (**i**) Inhibition of IL-33-induced mouse Raw cell migration by MAPK, p38 and IκBα specific inhibitors (*n*=6 samples per group). Vehicle-treated cells served as controls. (**j**) Inhibition of IL-33-induced Raw cell shape changes by MAPK, p38 and IκBα specific inhibitors (*n*=6 samples per group). Vehicle-treated cells served as controls. Arrowheads indicate filopodia sprouts of the IL-33-activated Raw cells. Scale bar, 50 μm (mean±s.e.m., Student's *t*-test). Full-gel images for **g**,**h** are shown in Supplementary Fig. 9.

As IL-33 was the most upregulated cytokine in PDGF-BB-stimulated pericytes, we investigated its functional impact on macrophages. FACS and RT–PCR analyses showed that Raw macrophage-like cell line expressed ST2 receptor that mediates biological functions of IL-33 (Fig. 4a). As a negative control, stromal fibroblasts lacked a detectable level of ST2 expression (Fig. 4a). Knowing that macrophages expressed ST2 receptor, we studied the functional impact of IL-33 on macrophages. Recombinant IL-33 stimulated Raw cell migration in a dose-dependent manner (Fig. 4b,c). In addition, IL-33 induced activated morphological changes of Raw cells that manifested an elongated cell shape (Fig. 4d). Importantly, the *St2*-siRNA knockdown significantly ablated the IL-33-induced Raw cell migration and morphological changes (Fig. 4c,d). To validate the biological effects of pericyte-derived IL-33 in modulation of macrophage activities *in vitro*, PDGF-BB-stimulated and non-stimulated pericytes were co-cultured with Raw cells. In this co-culture assay, PDGF-BB-stimulated pericytes induced elongated Raw cell morphological changes, which was neutralized by a soluble ST2 receptor (Fig. 4e). Similarly, PDGF-BB-primed pericytes also attract Raw cell motility in a co-culture system (Fig. 4f).

Consistent with the above-mentioned biological functions, IL-33 stimulation of Raw cells induced marked activation of MAPK, which became hyper-phosphorylated (Fig. 4g). In addition, IL-33 stimulated phosphorylation of p38 that is known in the regulation of cellular actin reorganization and cell morphological changes^19^ (Fig. 4g). Notably, IL-33 stimulation led to potent activation of IκBα, which became highly phosphorylated (Fig. 4g). However, Akt levels in IL-33-stimulated and non-stimulated cells remained unchanged (Supplementary Fig. 4a). To functionally link the ST2 receptor and IL-33-activated intracellular signalling components, we used the *St2*-siRNA knockdown technique. IL-33-induced MAPK, p38 and IκBα phosphorylation in Raw cells were largely inhibited by the *St2*-specific siRNA (Fig. 4h), indicating that IL-33 induces the ST2-dependent activation of these intracellular signalling components in macrophages. In contrast to IL-33, PDGF-BB lacked abilities to activate MAPK, p38 and IκBα, supporting the conclusion that PDGF-BB does not directly act on macrophages.

To define the biological functions of the IL-33-ST2-activated downstream signalling molecules, we treated the IL-33-stimulated Raw cells with various inhibitors that blocked the activation of a specific signalling component. As expected, the MAPK, p38 and IκBα inhibitors^20^,^21^,^22^ effectively blocked IL-33-stimulated phosphorylation of these intracellular signalling molecules (Supplementary Fig. 4b). Treatment with a known MAPK inhibitor (U0126) completely abolished IL-33-induced Raw cell migration and cell shape changes (Fig. 4i,j). These findings reconcile with the known functions of MAPK signalling. Similarly, p38 inhibitor also effectively inhibited IL-33-induced migration and cell shape changes of Raw cells (Fig. 4i,j). The treatment of IL-33-stimulated Raw cells with an NF-κB inhibitor also significantly attenuated IL-33-induced Raw cell migration and cell shape changes (Fig. 4i,j). These findings demonstrate that IL-33 displays direct effects on Raw cell migration and activation through MAPK, p38 and IκBα signalling pathways.

### IL-33 increases TAMs in tumours

**Figure 5:**
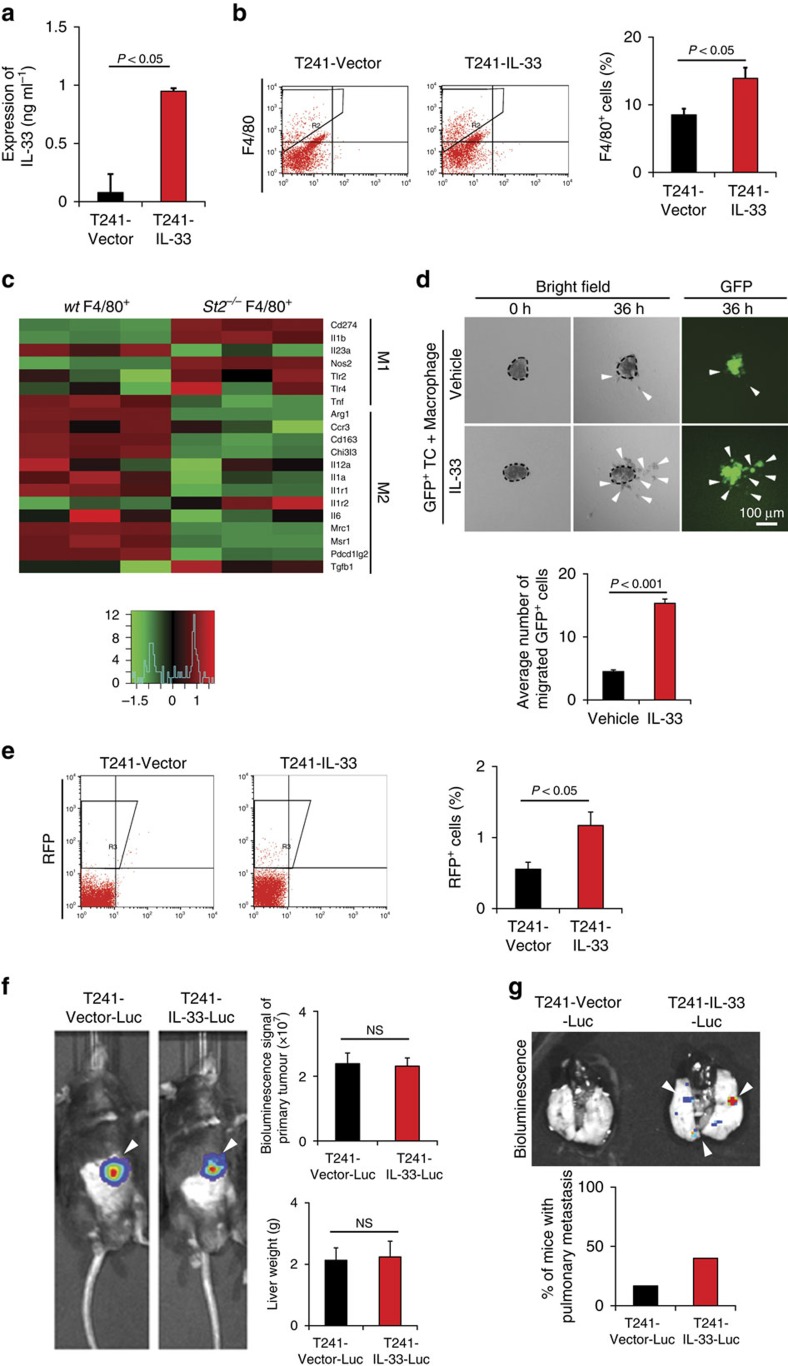
IL-33 induces infiltration of M2-like TAMs and metastasis. (**a**) IL-33 expression levels in vector- and IL-33-T241 tumour xenografts (*n*=6 samples per group). (**b**) FACS analysis of the total F4/80^+^ macrophages in vector- and IL-33-T241 tumour tissues (*n*=5 samples per group). (**c**) Heatmap of M1 and M2 related genes by genome-wide expression profiling of F4/80^+^ cells isolated from Panc02 tumour grafts implanted in *wt* and *St2^−/−^* mice (*n*=3 samples per group). (**d**) *In vitro* matrigel invasion of GFP^+^ LLC tumours in the presence of IL-33 or vehicle-stimulated macrophages (*n*=6 samples per group). Arrowheads point to spread GFP^+^ tumour cells. Scale bar, 100 μm. (**e**) FACS analysis and quantification of RFP^+^ circulating tumour cells in the peripheral blood of vector- and IL-33-T241 tumour-bearing mice (*n*=5 samples per group) at the time point of the average tumour size of 1.5 cm^3^. (**f**) Bioluminescent imaging of tumour-bearing mice implanted in livers with luciferase^+^ vector- and IL-33-T241 tumours. Arrowheads point to luciferase^+^ tumours. Quantifications of bioluminescence signals and liver weights (*n*=5 samples per group). NS, not significant. (**g**) Bioluminescent imaging of lungs of luciferase^+^ vector- and IL-33-T241 tumour-bearing mice. Arrowheads point to luciferase^+^ metastatic nodules. Quantifications of luciferase^+^ pulmonary metastases (*n*=8 animals per group; mean±s.e.m., NS, not significant, Student's *t*-test).

We performed *in vivo* gain-of-function experiments that allowed studying the effect of IL-33 on tumour growth, TAM recruitment and tumour invasion and metastasis. Genetic propagation of T241 tumours with IL-33 by lentiviral approach led to overexpression of IL-33 protein in tumour tissues (Fig. 5a). Despite a high level of IL-33 expression, IL-33-overexpressing tumours grew at similar rates *in vitro* and *in vivo* as vector-transfected control tumours (Supplementary Fig. 5a and b). However, FACS analysis showed that the number of F4/80^+^ TAMs in IL-33-tumours was markedly increased as compared with that of vector control tumours (Fig. 5b).

To further define the TAM phenotypes in tumours, we implanted IL-33 positive tumours in *wt* and *St2^−/−^* knockout mice^23^ and isolated TAMs from these tumours. The isolated primary TAMs from *wt* and *St2^−/−^* backgrounds were subjected to genome-wide affymetrix analysis. Intriguingly, classical M2 markers including *Cd206* (*Mrc1*), *Cd163*, *Pdl2* (*Pdcd1lg2*), *Ccr3*, *Arg1* and many others were all markedly downregulated in TAMs isolated from the *St2^−/−^* background as compared with those isolated from tumours grown in *wt* mice (Fig. 5c and Supplementary Fig. 5c).

We further investigated subpopulations of TAMs using M2 markers by FACS analysis in vector- and IL-33- overexpressed tumours. For defining the M2 population of macrophages, three independent cell surface markers including CD206, CCR3 and PDL2 were used in our FACS analysis. Altogether, three independent analyses show that F4/80^+^CD206^+^, F4/80^+^CCR3^+^ and F4/80^+^PDL2^+^ M2 subpopulations of macrophages were significantly increased (Supplementary Fig. 5d–f). However, the F4/80^+^CD206^−^ subpopulation was also significantly increased.

### IL-33-primed macrophages promote metastasis

One of the important characteristics of M2 macrophages is tumour promotion through various processes like metastasis. Since our study indicated increased tumour metastasis linked to IL-33, we investigated further the link between IL-33-induced TAMs and tumour metastasis. TAMs have been described to facilitate tumour cell invasion, intravasation and dissemination^1^,^24^. To functionally link IL-33-primed TAMs with cancer invasion, we performed *in vitro* matrigel cancer invasion experiments in which tumour cells and macrophages were co-embedded in matrigel as spheroids. IL-33-treated and non-treated macrophages were mixed with GFP^+^ LLC tumour cells and spreading of GFP^+^ cells was quantitatively measured. IL-33-stimulated macrophages, but not non-stimulated cells significantly promoted cancer cell invasion in this *in vitro* invasion assay (Fig. 5d). These findings show that IL-33-primed macrophages promote cancer cell invasion and possibly metastasis.

We next analysed circulating tumour cells (CTCs) in IL-33 and vector tumour-bearing mice. Interestingly, a significantly higher number of CTCs were found in IL-33-tumour-bearing mice as compared with vector tumour-bearing mice (Fig. 5e). To provide further evidence of IL-33-induced metastasis, we developed an independent metastasis model in which luciferase-expressing primary tumours were implanted in the liver of each mouse. Although no differences of primary tumour growth were observed (Fig. 5f), a higher number of IL-33-tumour-bearing mice developed luciferase^+^ pulmonary metastasis as compared with the vector control group (Fig. 5g). These findings were consistent with increased CTCs in IL-33-tumour-bearing mice and showed that IL-33 induced tumour cell intravasation and metastasis without affecting primary tumour growth.

### The IL-33-ST2-TAM axis-dependent metastasis

**Figure 6:**
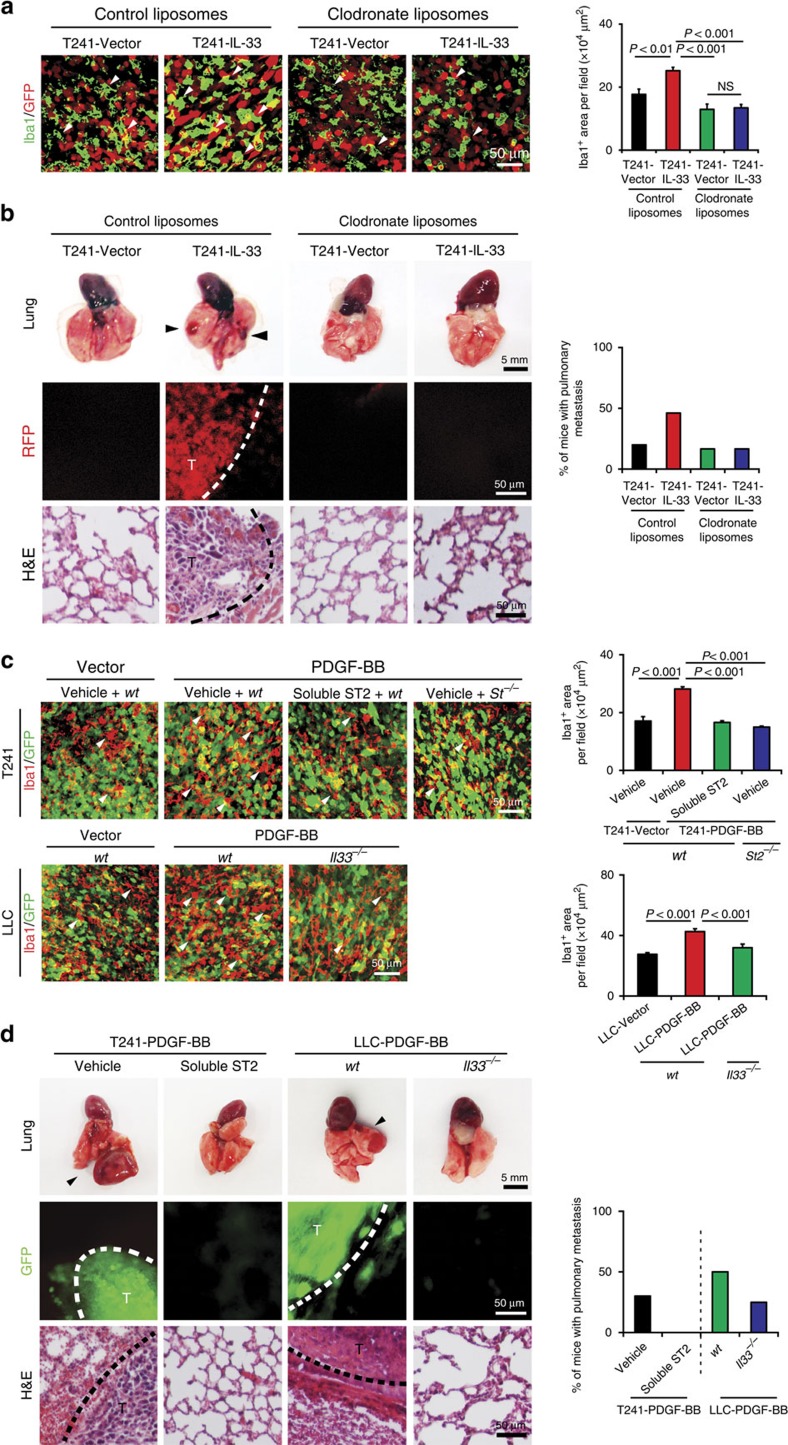
IL-33 mediates PDGF-BB-stimulated cancer metastasis through a TAM-dependent mechanism. (**a**) Clodronate effectively inhibited Iba1^+^ macrophage infiltration (green) in IL-33-T241 tumours. Tumour cells were labelled with RFP (red). Arrowheads indicate TAMs. Quantification of Iba1^+^ macrophage in clodronate-treated and non-treated vector- and IL-33-T241 tumours (*n*=8 random fields per group). (**b**) Lung metastasis in clodronate-treated and non-treated vector- and IL-33-T241 tumour-bearing mice. Arrowheads indicate lung surface metastatic nodules. Dashed line marks the border between the RFP^+^ metastatic nodule and surrounding lung tissues. T, tumour. Quantification of percentage of animals with pulmonary metastasis on the surface of lungs (*n*=10–16 mice per group). (**c**) Detection of Iba1^+^ macrophages (red) and tumour cells (green) in vehicle- and soluble ST2-treated PDGF-BB-T241 tumours implanted in *wt* mice. Iba1^+^ macrophages (red) were also detected in PDGF-BB-T241 tumours implanted in *St2^−/−^* mice. Vector-T241 tumour serves as a control. The detection of Iba1^+^ macrophages (red) and tumour cells (green) in PDGF-BB-LLC tumours implanted in *wt* and *Il33^−/−^* mice. Vector-LLC tumour serves as a control. Arrowheads indicate Iba1^+^ macrophages. Quantification of Iba1^+^ macrophages (*n*=8 random fields per group). (**d**) Pulmonary metastasis in vehicle- and soluble ST2-treated PDGF-BB-T241 tumour-bearing mice. Pulmonary metastasis in PDGF-BB-LLC tumour-bearing *wt* and *Il33^−/−^* mice. Metastases were detected by gross examination of lung surface, fluorescent detection for GFP^+^ signals and histological staining with H&E. Arrowheads indicate lung and liver surface metastases in tumour-bearing mice. Dashed lines encircle the borders between tumour nodules and surrounding tissues. T, tumour. Quantification of lung surface metastases in tumour-bearing mice (*n*=8–10 mice per group; mean±s.e.m., NS, not significant, Student's *t*-test).

To mechanistically link TAMs and cancer metastasis of IL-33-tumours, we used clodronate liposomes^25^ as macrophage-ablating agent to deplete TAMs. Expectedly, clodronate effectively ablated TAM numbers in both vector and IL-33-tumours (Fig. 6a). In contrast, the treatment of tumour-bearing mice with the control liposome did not significantly affect macrophage numbers in IL-33 tumours as compared with controls. Approximately 50% of IL-33 tumour-bearing mice possessed visible pulmonary metastatic nodules on the surface of their lungs at week 6 after removal of primary tumours (Fig. 6b). Conversely, the vector tumour control group had a lower rate of pulmonary metastasis. The lung metastatic lesions were validated by detection of red fluorescent protein (RFP; Fig. 6b). The depletion of TAMs by clodronate markedly decreased the metastatic incidences in the lungs of IL-33 tumour-bearing mice, whereas the low metastatic rate in vector-tumour bearing mice remained unchanged. These findings show that IL-33-promoted pulmonary metastasis is dependent on TAMs and the low metastatic incidence in control tumour-bearing mice might be mediated through a TAM-independent mechanism.

To further investigate the TAM-mediated metastatic potentials, we used a zebrafish metastasis model^26^,^27^,^28^ that allowed detection of the interactions between malignant cells and macrophages at the single-cell level. This zebrafish metastasis model also permits kinetic monitoring of tumour cell invasion and metastasis in the living fish body. Moreover, the availability of certain genetic strains such as transgenic Fli1:EGFP zebrafish^29^,^30^ allows us to study the event of tumour cell intravasation with or without co-injection of macrophages. Interestingly, the implantation of IL-33 tumour cells alone in the perivitelline space did not significantly display high dissemination (Supplementary Fig. 6a and b). However, co-implantation of IL-33 tumour cells and macrophages resulted in massive tumour cell dissemination from the primary sites and distal metastasis. Interestingly, a substantial number of IL-33 metastatic tumour cells in distal regions of the zebrafish body including the head and truck regions were coupled with co-injected macrophages, suggesting that tumour cells hijacked IL-33 stimulated macrophages for intravasation and dissemination. The injected macrophages in vector control tumours also significantly, albeit modestly, promoted tumour cell dissemination (Supplementary Fig. 6a and b). In another experimental setting, macrophages were stimulated with IL-33 protein *in vitro* before implantation with tumour cells *in vivo*. Again, the IL-33-educated macrophages significantly increased tumour cell invasion in this zebrafish model (Supplementary Fig. 6c and d), indicating that IL-33-activated macrophages play a critical role in cancer metastasis.

To encircle the functional loop between the PDGF-BB-PDGFRβ and IL-33-ST2 signalling pathways in the TAM-associated cancer invasion and metastasis, we took both pharmacological and genetic approaches to execute the IL-33 loss-of-function experiments in PDGF-BB tumours. For pharmacological blocking of IL-33 functions, we treated PDGF-BB and vector tumour-bearing mice with a soluble ST2 receptor, which have been used to effectively block IL-33 functions in other experimental settings^31^. Notably, treatment of PDGF-BB tumours with the soluble ST2 completely blocked the PDGF-BB-elevated Iba1^+^ TAMs that returned to the vehicle-treated control level (Fig. 6c). Similarly, PDGF-BB-expressing LLC tumours grown in *Il33^−/−^* mice also showed significantly decreased infiltration of TAMs that reached to a similar level of vector tumour grown in *wt* mice (Fig. 6c). Furthermore, similar reduction of TAMs in PDGF-BB tumours was also seen in *St2^−/−^* deficient mice (Fig. 6c). Collectively, these data show that the IL-33–ST2 signalling pathway mediates PDGF-BB-recruited TAMs in the tumour microenvironment.

Consistent with reduction of TAMs, treatment of PDGF-BB tumours with the soluble ST2 blocked PDGF-BB-promoted pulmonary metastasis (Fig. 6d). To further validate these findings, we studied PDGF-BB-promoted cancer metastasis in *Il33^−/−^* mice^32^. Primary PDGF-BB tumour growth was not altered in *wt* and *Il33^−/−^* mice (Supplementary Fig. 6e). However, PDGF-BB tumour-bearing *Il33^−/−^* mice showed attenuated metastasis as compared with PDGF-BB tumour-bearing *wt* mice (Fig. 6d). The presence of pulmonary metastatic lesions was further validated by GFP positivity. These findings provide compelling evidence that the IL-33–ST2 signalling pathway mediates PDGF-BB-triggered cancer metastasis.

### Endogenous IL-33 recruits TAMs and promotes metastasis

**Figure 7:**
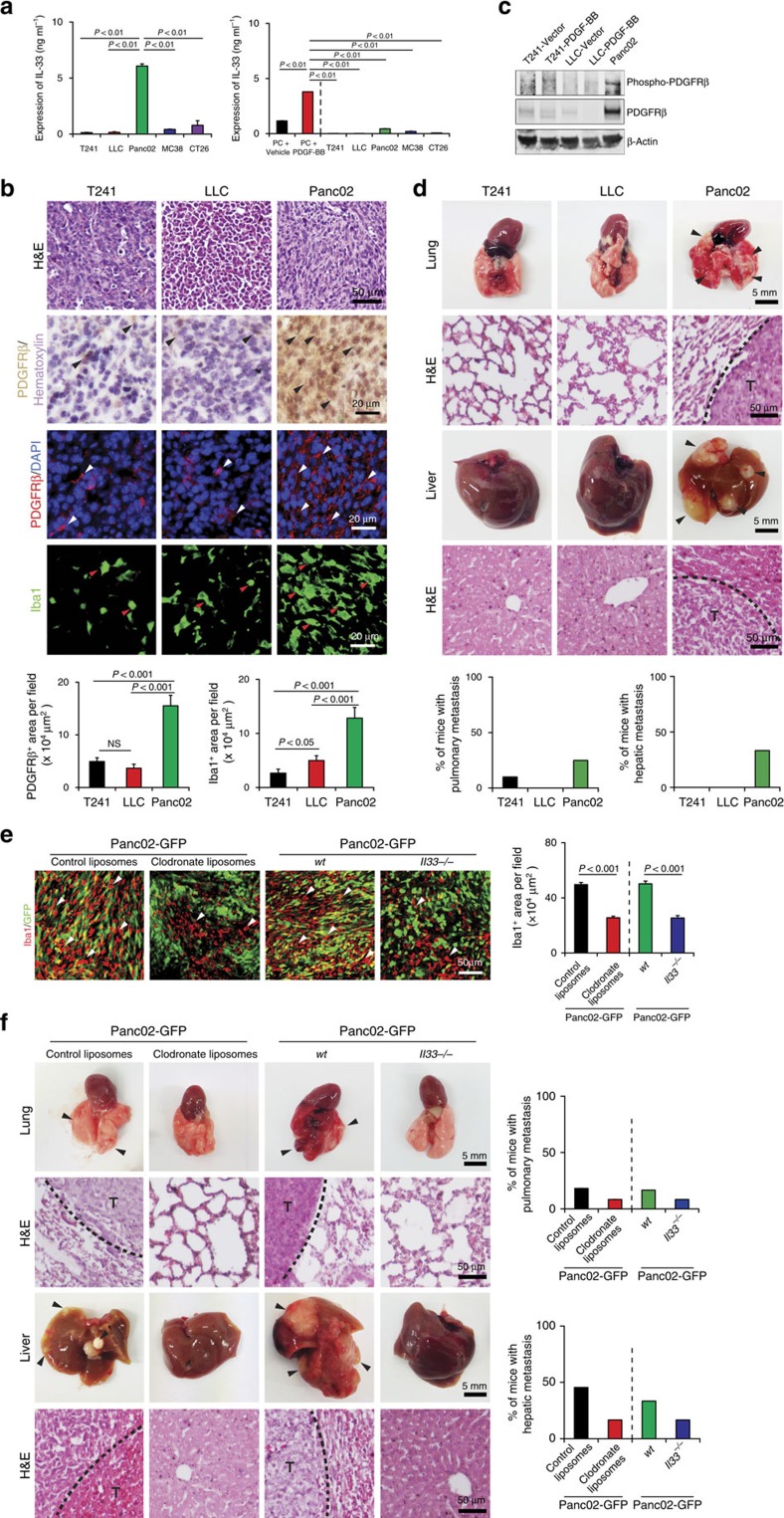
IL-33-recruited macrophages mediate cancer metastasis in a pancreatic tumour model. (**a**) ELISA detection of IL-33 protein expression levels in various xenograft tumour tissues and in cultured tumour cell lines (*n*=6 samples per group). PDGF-BB-stimulated and non-stimulated pericytes served as positive controls. (**b**) Immunostaining of PDGFRβ^+^ signals and Iba1^+^ macrophages in T241, LLC and Panc02 tumours. Upper two panels were counter-stained with H&E, or PDGFRβ and haematoxylin. Lower middle panels were double stained with DAPI (blue). Black and white arrowheads point to PDGFRβ^+^ signals. Red arrowheads indicate Iba1^+^ macrophages. Quantification of PDGFRβ^+^ signals and Iba1^+^ macrophages (*n*=8 random fields per group). NS, not significant. (**c**) Immunoblotting detection of total and phosphorylated PDGFRβ in Panc02 tumour tissues. Vector- and PDGF-BB-T241 or -LLC tumour tissues were used as controls. (**d**) Pulmonary and hepatic metastasis in *wt* T241-, LLC- and Panc02-tumour-bearing mice. Metastases were detected by gross examination of lung surface and liver surface, and histological staining with H&E. Arrowheads indicate lung and liver surface metastases in Panc02 tumour-bearing mice. Dashed lines encircle the borders between tumour nodules and surrounding tissues. T, tumour. Quantification of lung and liver surface metastases in tumour-bearing mice (*n*=8–10 mice per group). (**e**) Detection of Iba1^+^ macrophages (red) and tumour cells (green) in clodronate- and vehicle-treated Panc02-GFP^+^ tumours implanted in *wt* mice. Iba1^+^ macrophages (red) were also detected in Panc02-GFP^+^ tumours implanted in *Il33^−/−^* mice. Wild type of mice serves as a control. Arrowheads indicate Iba1^+^ macrophages. Quantification of Iba1^+^ macrophages (*n*=8 random fields per group). (**f**) Pulmonary and hepatic metastasis in clodronate- and vehicle-treated Panc02-GFP^+^ tumour-bearing mice, and *wt* or *Il33^−/−^* mice with Panc02-GFP^+^ implantation. Metastases were detected by gross examination of lung surface and liver surface, and histological staining with H&E. Arrowheads indicate lung and liver surface metastases in tumour-bearing mice. Dashed lines encircle the borders between tumour nodules and surrounding tissues. T, tumour. Quantification of lung and liver surface metastases in tumour-bearing mice (*n*=8–10 mice per group; mean±s.e.m., NS, not significant, Student's *t*-test). Full-gel images for **c** are shown in Supplementary Fig. 9.

To relate our findings to pathophysiological relevance, we analysed IL-33 expression levels in various tumour tissues. We found that the Panc02 pancreatic xenograft tumour expressed endogenous IL-33 at a high level (>6,000 pg ml^−1^) as compared with other tumour types (Fig. 7a). Surprisingly, the analysis of IL-33 expression in cultured Panc02 cells *in vitro* showed only a modest expression level (<500 pg ml^−1^), although this level was higher than other cultured tumour cells (Fig. 7a). High expression of IL-33 *in vivo* tumour tissues but not *in vitro* cultured Panc02 tumour cells indicated that host cellular components in tumour tissues contributed to IL-33 expression. We therefore analysed tumour tissues and found that Panc02 tumour tissues contained an extremely high proportion of the stromal component that constituted the majority of the tumour tissues (Fig. 7b). In contrast, other tumour tissues including those of T241 fibrosarcoma and LLC lung cancer possessed only little stromal components (Fig. 7b). These findings are in general agreements with pancreatic cancers that contain high stromal cellular components, which are correlated with an invasive phenotype^33^.

We localized PDGFRβ expression in various tumour xenografts and found that the Panc02 tumour expressed PDGFRβ at a high level as compared with other tumour types (Fig. 7b). Moreover, PDGFRβ expression was restricted to stromal fibroblast components and Panc02 tumour cells *in vitro* have barely detectable levels of PDGFRβ expression (Supplementary Fig. 7a), supporting the non-tumour cell expression of PDGFRβ. Phosphorylation analysis showed that a substantial proportion of PDGFRβ molecules were phosphorylated in Panc02 tumour tissue (Fig. 7c). PDGF-BB is a known and potent ligand for the activation of PDGFRβ^34^. However, PDGF-BB was barely detectable in Panc02 tumour cells (Supplementary Fig. 7b), suggesting an alternative mechanism for the PDGFRβ activation, probably through a receptor autophosphorylation mechanism owing to the formation of receptor dimers or oligomers^35^,^36^,^37^. Consistent with the mouse IL-33 fibrosarcoma model, Panc02 tumours also contained an exceptionally high number of TAMs as compared with other tumour types (Fig. 7b), validating the causational relation between IL-33 and TAM recruitment.

In a subcutaneous xenograft model, Panc02 tumour-bearing mice manifested haematogenous metastasis in several organs including lung and liver (Fig. 7d). Notably, liver metastasis was the dominant route for Panc02 tumour spreading, whereas pulmonary metastatic nodules were occasionally detectable (Fig. 7d). These findings demonstrate that the Panc02 pancreatic tumour is a highly invasive and metastatic cancer type.

### TAM-dependent metastasis of high IL-33 tumours

To define the causational relation between TAMs and Panc02 metastasis, Panc02 tumour-bearing mice were treated with clodronate liposomes to deplete TAMs. Expectedly, clodronate treatment significantly ablated the total number of TAMs in Panc02 tumour tissues (Fig. 7e). Similarly, Panc02 tumours grown in *Il33^−/−^* mice contained a significantly less number of TAMs as compared with those tumours grown in *wt* mice as measured by immunohistochemistry and FACS (Fig. 7e and Supplementary Fig. 7c). Consistently, significant reduction of IL-33 expression in Panc02 tumours was detected in *Il33^−/−^* mice (Supplementary Fig. 7d), supporting the fact that host cellular components are main sources of IL-33 production. Importantly, both pulmonary and liver metastases were markedly inhibited in clodronate-treated and *Il33^−/−^* deficient tumour-bearing mice (Fig. 7f). Finally, multiple data set network analyses of human tissues from Genemania^38^ showed that expression of *Pdgfrb* and *Il33* are positively co-localized (Supplementary Fig. 7e), supporting the existence of a regulatory pathway in humans as seen in mice. These data further strengthen our conclusions that IL-33-induced TAMs are largely responsible for metastasis.

**Figure 8:**
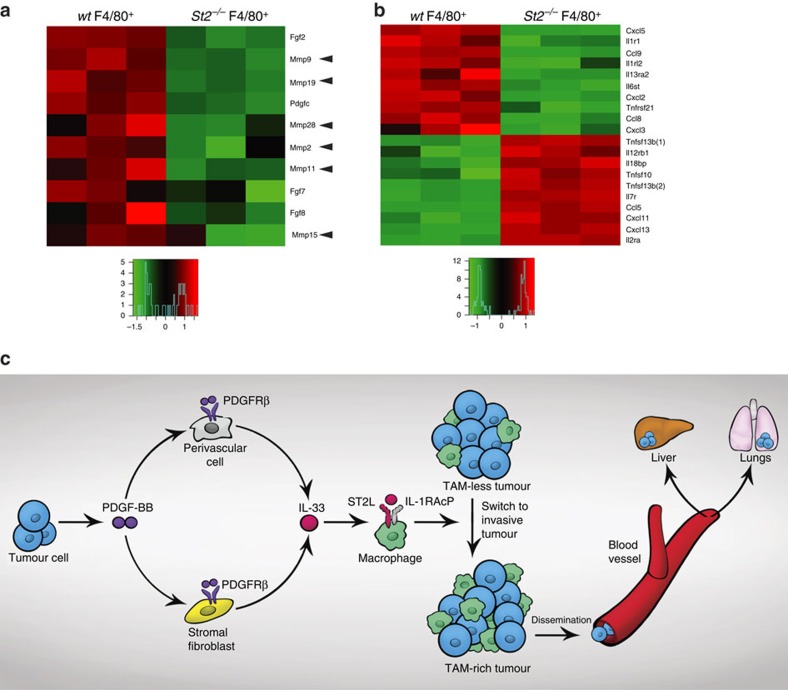
Genome-wide profiling of *wt* and *St2^−/−^* TAMs and survival correlation of IL-33 expression in grafted tumours. (**a**) Heatmap of top 10 metastasis-related genes by genome-wide expression profiling of F4/80^+^ cells isolated from Panc02 tumours implanted in *wt* and *St2^−/−^* mice (*n*=3 samples per group). (**b**) Heatmap of top 10 up- and top 10 down-most cytokines, chemokines and their receptor genes by genome-wide expression profiling of F4/80^+^ cells isolated from Panc02 tumours implanted in *wt* and *St2^−/−^* mice (*n*=3 samples per group). (**c**) Schematic diagram shows in pericytes and stromal fibroblasts the PDGF-BB-PDGFRβ-IL-33-ST2 axis-recruited macrophages in switching a noninvasive tumour to a highly invasive tumour.

We next analysed gene expression profiles of isolated TAMs from Panc02 tumours grown in *wt* and *St2−/−* mice. Interestingly, *Cd206* (*Mrc1*) was among the top 10 downregulated genes, indicating loss of the M2 phenotype of macrophages in *St2^−/−^* TAMs (Supplementary Fig. 8a). Macrophage metastasis-related genes including matrix-degradation proteases, angiogenic factors and direct tumour invasion effectors were analysed^39^. Interestingly, among 56 metastasis-related gene products, many matrix metalloproteinases (MMPs) including *Mmp2, Mmp9, Mmp11, Mmp15, Mmp19* and *Mmp28* are among the top 10 downregulated genes in *St2^−/−^* TAMs (Fig. 8a). These findings suggest that TAMs possibly promote cancer invasion through the production of MMPs. In addition, we also performed genome-wide gene expression analysis of cytokines, chemokines and their receptors. However, both upregulation and downregulation of these cytokines were found (Fig. 8b). Notably, *Ccl2* expression was not altered between *St2^+/+^* and *St2^−/−^* TAMs. Similarly, *Ccl2* expression is not decreased in Panc02 tumours implanted in *St2^−/−^* and *Il33^−/−^* mice compared with those in *wt* mice as validated by qPCR (Supplementary Fig. 8b). Also, chemokine receptors were not among top regulated genes between *St2^+/+^* and *St2^−/−^* TAMs. These data suggest that the CCL2-CCR signalling is less likely involved in mediating TAM-induced metastasis in our model system. However, we cannot completely exclude the possibility of involvement of chemokines and their receptors in recruiting TAMs.

## Discussion

The current work was initiated by our original surprising finding that PDGF-BB-expressing tumours contained a high number of TAMs that lacked PDGFR expression. We therefore asked a crucial mechanistic question: Through what mechanism does PDGF-BB recruit macrophages? The fact that monocytes and macrophages lack detectable PDGFR expression implies the existence of an indirect mechanism that underlies macrophage recruitment by PDGF-BB in the tumour microenvironment. To uncover this indirect mechanism, we first analysed possible receptor types that mediate PDGF-BB-induced TAM recruitment and demonstrated that PDGFRβ, but PDGFRα, is the crucial receptor mediating PDGF-BB-recruited TAMs. PDGFRβ is primarily expressed in perivascular cells and stromal fibroblasts as epithelial LLC cancer cells completely lack PDGFRβ expression^7^. Thus, perivascular cells and stromal fibroblasts should be the primary cell types responsible for TAM recruitment.

Pericytes as the main perivascular cell type often exist in tumour microvasculatures^5^,^7^,^40^ and their functions in tumour growth, invasion and metastasis are largely unknown. Coverage of microvessels with pericytes increases maturation and stability of tumour vessels that would potentially support tumour growth^41^. Conversely, pericyte coverage of tumour vessels may prevent intravasation of tumour cells into the circulation and thus decreases metastatic potentials^42^. Therefore, mechanisms of tumour vasculature-associated pericytes in tumour growth and metastasis may be complex and somehow paradoxical. In general, molecular mechanisms of pericyte-derived signalling molecules in modulation of the tumour microenvironment are overlooked in the field of cancer research. To date, most studies focus on characterization of signalling molecules that affect pericyte proliferation, migration and morphological changes^41^. Unlike most other studies, we have taken a genome-wide approach to define pericyte-derived signalling molecules that affect behaviour and functions of other cellular components in the tumour microenvironment. One of the most striking discoveries of our present study is that IL-33 is the most upregulated gene product in the whole genome of PDGF-BB-stimulated pericytes. This is an unexpected discovery because PDGF-BB is known to stimulate pericyte proliferation and migration. Thus, gene products involving in cell division, motility and cytoskeleton reorganization would be expected to be within the top-listed genes of PDGF-BB-stimulated pericytes. Further, the PDGF-BB-induced IL-33 expression is also observed in stromal fibroblasts, indicating the existence of a generally regulatory mechanism of the PDGF-BB-PDGFRβ-IL-33 axis. In contrast to pericytes and stromal fibroblasts, vascular endothelial cells isolated from PDGF-BB-positive tumours did not show elevated expression levels of IL-33. However, endothelial cells have been described as the major cellular source of IL-33 in chronically inflamed tissues under other pathological conditions such as rheumatoid arthritis and Crohn's disease^43^. Perhaps, the cellular sources of IL-33 are different under different pathophysiological conditions. We provide mechanistic data to demonstrate that the PDGF-BB-PDGFRβ signalling pathway modulates the IL-33 promoter activity through the SOX7-mediated transcriptional regulation. This seems to be a generalized regulatory mechanism existing in PDGFRβ^+^ cells. The exceptionally high level of IL-33 in PDGF-BB-stimulated pericytes suggests the existence of a novel functional pathway since IL-33 is a relatively newly identified cytokine. Despite its known functions in the regulation of immune responses^12^,^14^,^23^,^32^, the role of IL-33 on monocytes/macrophages is relatively unexplored. Particularly, the IL-33-triggered signalling in the tumour microenvironment in relation to inflammation-associated tumour invasion is unknown.

We showed that monocytes and macrophages express ST2 receptor, which becomes activated in response to IL-33 stimulation. The interaction between IL-33 and ST2 is functionally meaningful as downstream signalling components such as MAP kinase become activated, leading to macrophage migration. IL-33-induced migratory effect could be important for the recruitment of TAMs in tumours from peripheral tissues such as those in surrounding tissues and peripheral blood. TAMs showed a M2-like phenotype characterized by expression of *Cd206* (*Mrc1*), *Cd163*, *Pdl2* (*Pdcd1lg2*), *Chi3i3, Arg1*, as well as tumour-promoting molecules involved in invasion and metastasis like *MMPs*.

The next question is what IL-33-stimulated TAMs do for tumour growth and invasion. To address this important functional issue, we have taken both gain-of-function and loss-of-function approaches. Overexpression of IL-33 in tumours has no impact on primary tumour growth. However, IL-33 triggers extensive haematogenous metastasis, which is dependent on TAMs. These findings are in general agreement with TAM functions, especially the CD206^+^ M2 macrophages^44^ that facilitate tumour invasion and metastasis. Although TAMs might affect several steps of the metastatic cascade, the IL-33-stimulated TAMs are likely to increase intravasation of tumour cells into the circulation. At this early stage of metastasis, TAMs may guide tumour cells to transmigrate through the vessel wall by interacting with the endothelial cells. Again, this is another example how tumour cells manipulate various host cells for invasion and metastasis. In contrast to our findings, a recent study shows that systemic injection of IL-33 stimulates primary tumour growth and metastasis in a mouse tumour model^16^. At this time of writing, the difference between our findings and this study is unclear. It is plausible that systemic delivery of IL-33 protein in mice as shown in that study could elicit a broad immune response that favour tumour growth. Consequently, IL-33-accelerated tumour growth rates are also coupled to increased metastasis. Thus, in that study, it is unclear whether IL-33-associated metastasis is owing to large tumour sizes or other mechanisms. Two published studies also show that IL-33 exhibits antitumour activity through modulation of cytolytic T cells and NK cells^45^,^46^. In addition, ‘alarmin' IL-33 may also act as an immunoadjuvant to inhibit tumour growth^47^. Although these findings are primarily focused on the effect of IL-33 on primary tumour growth, our data show that IL-33 promotes metastasis through a distinct mechanism by which metastasis occurs through a primary tumour size-independent mechanism.

Taken together, our present work not only defines a novel mechanistic pathway of host cells in the tumour microenvironment that controls cancer metastasis, but also indicates that TAMs are the primary cell types that governs the metastatic process (Fig. 8c). Targeting the PDGF-BB-PDGFRβ-IL-33–ST2 signalling axis in the stromal compartment would provide a novel therapeutic option for the treatment of cancer and metastasis.

## Methods

### Cell culture

PDGF-BB- and vector-transfected T241 fibrosarcoma and LLC stable cell lines were established by a Murine Stem Cell Virus (MSCV) vector containing enhanced green fluorescent protein (GFP). The human A431 epidemoid carcinoma cell line was kindly provided by Dr Keiko Funa from the Gothenburg University, Sweden. The shRNA-*Pdgfb*-containing lentivirus (HSH012856, GeneCopoeia) was amplified in 293 T cells. The infected EGFP^+^ A431 cells were sorted by FACS and shRNA efficiency was detected by qPCR. Murine pancreatic cancer cell line Panc02 was kindly provided by Dr Maximilian Schnurr from University of Munich, Germany. The S17 stromal cells were cultured in a Minimum Essential Medium Alpha Medium supplemented with 10% fetal bovine serum (FBS)^48^. Mouse monocyte/macrophage RAW 264.7 cell line was kindly provided by Dr Martin Rottenberg from the Karolinska Institutet, Sweden. FACS sorting was used to isolate primary NG2^+^ pericytes (NG2 antibody: Catalogue (Cat.) No. AB5320, Millipore) from mouse healthy lung tissues and F4/80^+^ TAMs (F4/80 antibody: Cat No.123122, Biolegend) from mouse tumours. All other cell lines were grown and maintained in Dulbecco's modified Eagle's medium (DMEM) supplemented with 10% FBS. All the cell lines were not authenticated after purchase or transferred from other laboratories. We routinely tested mycoplasma contaminations in all our cell lines and they were negative.

### Chromatin immunoprecipitation assay

Mouse primary pericytes were used for the ChIP assay, which was performed according to the manufacturer's standard protocol using an EZ-ChIP kit (Cat. No. 17-371, Millipore). In brief, the cells were fixed with 4% paraformaldehyde (PFA) before sonication with an agarose-blocking buffer, followed by incubation overnight with 2.5 μg of a non-immune sheep IgG (Cat. No. 12-515, Millipore) or an anti-SOX7 antibody^49^ (kindly provided by Dr Valerie Kouskoff, Cancer Research UK Manchester Institute, United Kingdom) per immunoprecipitation reaction. To quantitatively analyse relative levels of precipitated chromatin, quantitative PCR was used with primers directed against specific fragments of interested genes. The SRY-box containing promoter fragment of mIL-33 was amplified using the following primers: forward 5′-TGCAAGAAGGCAAATGCTAC-3′; and reverse 5′-ATAGCTGACCTGCCTCCCTAC-3′. To amplify a control fragment lacking the SRY-box consensus sequence within the coding region of IL-33, the following primers were used: Forward 5′-CACTGATCTGGAAACTCGCAAC-3′; and reverse 5′-TTATAGCCTGGTCCTTCATCTC-3′. Fragment amplification in total input was used to adjust the enriched values after immunoprecipitation.

### Animals

All animal studies were approved by the North Stockholm Animal Ethical Committee. Female C57BL/6 and immunodeficient CB17/Icr-Prkdcscid/IcrCrl (SCID) mice were provided by the breeding unit of the animal facility of Department of Microbiology, Tumor and Cell Biology, Karolinska Institute, Sweden. The C57BL/6-*Il33^−/−^* mice were generated by Dr Susumu Nakae (University of Tokyo, Japan). The C57BL/6-*T1/ST2^−/−^* mice were generated as described^23^. Zebrafish of the Tg(*fli1:EGFP*)y1 (ZFIN, Eugene) was used for metastasis assay as described^29^,^30^.

### Xenograft tumour models and metastasis

Female 4- to 8-week-old C57/B6 or SCID mice were used. For most experiments, 1 × 10^6^ cells per 0.1 ml tumour cells were subcutaneously injected into the middle region of the dorsal back of each mouse. In a subset of experiments, tumour cells were stably transfected with a luciferase-expressing lentiviral vector. After creating an incision 0.5 × 10^6^ cells per 0.03 ml tumour cells were injected into the left liver lobe of each mouse. After tumour cell implantation, the incision was sutured. For subcutaneous tumour implantation experiments, the tumour size was measured every other day using a caliper and the tumour volumes were calculated by a standard formula: Volume=Length × Width × Width × 0.52 (ref. 50). For liver tumour implantation, the tumour sizes were monitored with an IVIS Spectrum CT system (PerkinElmer). Briefly, tumour-bearing mice were injected with D-luciferin (150 mg kg^−1^, PerkinElmer). Luminescence positive signals were detected by IVIS Spectrum CT system after 10–20 min injection (PerkinElmer). Subcutaneous primary tumours were surgically removed at the approximate size of 1.5 cm^3^. The mice were observed for 4–6 weeks for development of metastases. Once the mice were killed, the organs including liver and lungs were removed and surface metastases were photographed. Metastatic lesions were detected by haematoxylin and eosin (H&E) histological analysis and fluorescent microscopy.

### Drug treatment

For depletion of macrophages, 100 μl control or clodronate liposomes (dichloromethylene bisphosphonate; ClodronateLiposomes, The Netherlands) were intravenously injected every 4 days starting from 3 days before tumour implantation and continued until primary tumour removal. The mice were kept for an additional 4–6 weeks for the detection of metastases. For IL-33 neutralization *in vivo*, tumour-bearing mice were daily treated by subcutaneous injection with phosphate-buffered saline (PBS) or a soluble ST2 (sST2, 0.1 mg per mouse)^31^ starting from 1 day before tumour cell injection. After surgical removal of primary tumours, the treatment was terminated and the mice continued for metastasis experiments. For specifically neutralizing PDGFRs, an anti-PDGFRα (PDGFRα blockade, IH3, ImClone Inc.) or an anti-PDGFRβ (PDGFRβ blockade 2C5, ImClone Inc.) was intraperitoneally injected (0.8 mg per mouse) twice per week for 2 weeks. The tumours were collected for further experimentation. For imatinib (LC Laboratories, Woburn, MA, USA) treatment, the mice were orally administrated with imatinib (50 mg kg^−1^ daily). For metastasis experiments, imatinib treatment was terminated after primary tumour removal and the experiments were continued for 4–6 weeks. A lethal dose of CO_2_ was used to kill the animals.

### Whole-mount staining

The whole-mount protocols in our laboratory were used^25^,^51^,^52^. Briefly, fresh tumour tissues were fixed with 4% PFA at 4 °C overnight and the fixed tissues were cut into small pieces and digested with proteinase K (20 mM) in a Tris buffer, permeabilized with 100% methanol, washed and blocked overnight with 3% milk in 0.3% Triton X-100 in PBS. Primary antibodies against Iba1 (rabbit, Cat. No. 019-19741, WAKO), F4/80 (Rat, Cat. No. MCA497G, AbD Serotec), F4/80 (Rabbit, Cat. No. NBP2-12506, Novus Biologicals) and Ki67 (Rat, Cat. No. 14-5698-82, eBioscience) were incubated overnight at 4 °C, followed by washing, blocking with 3% milk and incubation with fluorescent-conjugated secondary antibodies for 2 h at room temperature. Additional washing was performed before mounting. The stained tissues were mounted with Vectashield mounting medium (Cat. No. H-1000, Vector Laboratories). Fluorescent signals were examined with a confocal microscope (Zeiss LSM510 Confocal, or Nikon C1 Confocal microscope) and quantitative analysis was performed with a Photoshop (CS5) software.

### Affymetrix gene-array analysis

The primary pericytes isolated from the lung tissues were treated with or without 100 ng ml^−1^ human PDGF-BB for 5 days and RNA samples were prepared using an RNAeasy kit (Qiagen) and hybridized using Affymetrix 1.0 ST Gene arrays. The sample preparation and analysis method for microarrays of PDGF-BB-treated pericytes is described as follows^53^. Triplicates of each sample were used for gene expression analysis. Normalization and analysis for differentially expressed genes are performed using robust multi-array analysis and significance analysis of microarrays (SAM) via R statistical software packages, oligo and samr. Heatmaps were presented for up- and downregulation of gene expression using the Multiple Experiment Viewer system (version 4.7). Survival data and gene expression data of uterine carcinosarcoma patients and kidney renal papillary cell carcinoma patients from The Cancer Genome Atlas (TCGA) is analysed for IL-33-high (above mean) and IL-33-low (below mean) groups. For breast cancer, the top 25% IL-33-high and lowest 25% IL-33-low groups were analysed. The statistical difference was analysed using Kaplan–Meier survival method followed by log-rank test.

### Stable expression of IL-33 in tumour cell lines

The full-length complementary DNA (cDNA) sequence coding human IL-33 was cloned into an expression vector, pLVX-IRES-tdTomato Vector (Cat. No. 631238, Clontech). Briefly, 293 T cells were transfected with the vector containing the cDNA sequence coding for human IL-33 using a Lenti-X HTX Packaging System (Clontech). Murine T241 fibrosarcoma cells were cultured with the filtered viral supernatants overnight. RFP^+^ cells were sorted by FACS and the IL-33 expression level was quantified by an ELISA (enzyme-linked immunosorbent assay) assay.

### Intracellular signalling inhibition and immunoblotting

Pericytes and macrophages were starved in 2% FBS-DMEM overnight, followed by pre-treatment with selective inhibitors against MEK1/2 (U0126, Cat. No. 1144, Tocris Bioscience), p38 (SB203580, Cat. No. 1202, Tocris Bioscience), IκBα (Withaferin A, Cat. No. 2816, Tocris Bioscience) or AKT1/2 (AKTi-1/2, Cat. No. ab142088, Abcam) for 1 h before stimulation with either IL-33 or PDGF-BB (50 ng ml^−1^) for 10 min or 24 h, and the cell lysates were collected for protein or RNA analyses.

The fraction of total cell lysate protein was prepared using a Triton X-100-based cell lysis buffer containing a cocktail of proteinase (Cat. No. 8340, Sigma) and phosphatase inhibitors (Cat. No. 5870, Cell Signaling). For protein separation, a standard molecular weight marker (Cat. No. 26616, Thermo Scientific) and an equal amount of protein from each sample were loaded on a SDS–PAGE (polyacrylamide gel electrophoresis) gel (Cat. No. NP0321/NP0323, Life Technologies), followed by transferring onto a nitrocellulose membrane (Cat. No. 88018, Thermo Scientific), which was subsequently blocked with 5% BSA (Cat. No. A7030-100MG, Sigma) for 30 min. Incubation with a specific primary antibody followed by an anti-mouse secondary antibody conjugated with IRDye 680RD (LI-COR, Lincoln, NE, USA; 1:15,000) or an anti-rabbit secondary antibody conjugated with IRDye 800CW (LI-COR, Lincoln, NE, USA; 1:15,000). Target proteins were detected using an Odyssey CLx system (LI-COR). Beta-actin was used as a control for all blots. The primary and secondary antibodies include: a mouse anti-mouse β-actin (Cat. No. 3700, Cell Signaling), a rabbit anti-mouse AKT (Cat. No. 9272, Cell Signaling), a mouse anti-mouse Phospho-AKT (Cat. No. 4051, Cell Signaling), a rabbit anti-mouse ERK (Cat. No. 4695, Cell Signaling), a rabbit anti-mouse Phospho-ERK (Cat. No. 9101, Cell Signaling), a rabbit anti-mouse IκBα (Cat. No. 4812, Cell Signaling), a rabbit anti-mouse Phospho-IκBα (Cat. No. 2859, Cell Signaling), a rabbit anti-mouse p38 (Cat. No. 9212, Cell Signaling) and a rabbit anti-mouse Phospho-p38 (Cat. No. 4631, Cell Signaling). Full-gel images are shown in Supplementary Figs 9 and 10.

### Phalloidin staining

Subconfluent monolayers of cells grown on cover slips were fixed with 4% PFA in PBS, followed by permeabilization with 0.1% Triton X-100. Cover glasses were inverted onto a drop of the fluorescent (TRITC) phalloidin conjugate (Cat. No. P1951, Sigma) in PBS and were incubated for 40 min at room temperature. The mounting of cover glasses onto slides was done using the Vectashield mounting medium containing DAPI (Cat. No. H-1200, Vector Laboratories).

### Co-culture

Macrophages were starved in 2% FBS overnight and seeded on coverslips. Prior to co-culture, pericytes were stimulated with PDGF-BB (50 ng ml^−1^) for 24 h. An equal number of pericytes and macrophages (1 × 10^4^ cells ml^−1^) were co-seeded in each well of a 24-well plate. Macrophages stimulated with recombinant IL-33 (50 ng ml^−1^) were served as a positive control. A soluble ST2 protein (200 ng ml^−1^) was added to block IL-33 function. Co-cultures were kept at 37 °C for 24 h before fixation with 4% PFA, permeabilization with 0.1% Triton X-100 in PBS, staining with a rabbit anti-mouse F4/80 antibody (Cat. No. NBP2-12506, Novus Biologicals), followed by incubation with a Alexa Fluor 488-labelled donkey anti-rabbit conjugated secondary antibody (Cat. No. A21206, Invitrogen). The cells were subsequently stained with TRITC-conjugated Phalloidin (50 μg ml^−1^. Cat. No. P1951, Sigma) and mounted with a Vectashield mounting medium containing DAPI (Cat. No. H-1200, Vector Laboratories) to visualize cell nuclei. Morphology was examined under a fluorescent microscope (Nikon Eclipse C1) at × 20 magnification.

### Matrigel invasion assay

GFP-labelled LLC tumour cells (1 × 10^4^ cells ml^−1^) were mixed with an equal number of macrophages and seeded onto low adhesion 96-well plates with rounded bottom (Nunc low cell binding MicroWell plate. Cat. No. Z721093-8EA, Sigma). Mixed cells were co-cultured for 24 h. Spheres were stimulated with 50 ng ml^−1^ IL-33 and mixed with growth factor reduced matrigel matrix (Cat. No. 354230, Corning) on ice before seeding into a 96-well plate. Bright field pictures were taken immediately after matrigel solidification (time point 0 h) using a light microscope. Spheres were subsequently incubated for 36 h before imaging (Nikon Eclipse C1) to distinguish GFP-labelled tumour cells. Matrigel-invading cells were manually counted using Photoshop software.

### RNAi experiments

Monolayers of cells cultured to about 70% confluency were subjected for siRNA transfection using the protocol as recommended by the manufacturer (Dharmacon). The following siRNAs (Dharmacon) were used: ON-TARGETplus SMARTpool siRNA against *Sox7* (Cat. No. L-044294-01-0005), *Il1rl1* (Cat. No. L-040418-01-0005) against *St2*, and scrambled control (Cat. No. D-001810-01-20). For transfection, siRNA at a final concentration of 25 nM with the DharmaFECT transfection reagent 1 (Cat. No. T-2001-02, Thermo Scientific) was used. The qPCR analysis was used to quantify the expression levels of siRNA-targeted genes.

### Chemotaxis assays

A modified Boyden chamber protocol was used to measure cell migration. Nitrocellulose membrane with the pore size of 8 μm was coated with 1% gelatine. The IL-33 chemo attractant at 50 ng ml^−1^ was diluted in medium and added to lower chambers. Typically, the cells were pre-incubated for 1 h with inhibitors before seeding to upper wells (3 × 10^4^ per well). Cell-containing chambers were incubated at 37 °C for 4 h. The cells were fixed with 100% methanol, followed by staining with Giemsa dye (Cat. No. GS500-500 ML, Sigma). The cells facing the upper chamber were stripped and the cell numbers facing the lower chambers were counted under a light microscope (Nikon eclipse, TS100). At least six samples per group were used for quantification and statistical analyses.

### Zebrafish metastasis model

Fertilized zebrafish (Danio rerio) eggs were incubated at 28 °C in Danieau's solution and cultivated under standard laboratory conditions. Zebrafish embryos at 24 h post fertilization were incubated with water containing 0.2 mM 1-phenyl-2-thio-urea (Sigma) to prevent pigmentation. At 48 h post fertilization, zebrafish embryos were dechorionated by a pair of sharp tip forceps and anaesthetized with 0.04 mg ml^−1^ of tricaine (MS-222, Sigma). Anaesthetized embryos were transferred onto an agarose gel mode for microinjection. The tumour cells and macrophages were labelled *in vitro* with 2 g ml^−1^ of 1,1-dioctadecyl-3,3,3,3-tetramethylindocarbocyanine perchlorate (DiI, Sigma, USA) and Vybrant DiD cell-labelling solution (DiD, Life Technologies, USA), respectively. The tumour cells (300–500 cells) were resuspended in 5 nl serum-free DMEM (Hyclone) were injected into the perivitelline space of each embryo using an Eppendorf microinjector (FemtoJet 5247, Eppendorf and Manipulator MM33-Right, Märzhäuser Wetziar). Non-filamentous borosilicate glass capillary needles were used for microinjection (1.0 mm in diameter, World Precision Instruments, Inc.). After injection, the fish embryos were immediately transferred into aquarium water containing 0.2 mM 1-phenyl-2-thio-urea. The injected embryos were kept at 28 °C and were examined at day 4 for monitoring tumour growth and invasion using a fluorescent microscope (Nikon Eclipse C1).

### ELISA assay

Tissue and *in vitro* cell samples lysed with a lysis buffer (Cat. No. 3228, Sigma), followed by 20-min centrifugation to remove cellular debris. Plasma samples were obtained from whole blood processed by collection into anti-coagulant-containing plasma tubes followed by 15-min centrifugation. Conditional media were collected at 48 or 72 h of confluent monolayer cells and were centrifuged to remove cellular debris before use. Four different assays were performed according to manufacturer's protocol to detect hPDGF-BB (Cat. No. DY220, R&D Systems), mPDGF-BB (Cat. No. MBB00, R&D Systems), mIL-33 (Cat. No. M3300, R&D Systems) or hIL-33 (Cat. No. 435907, BioLegend). Optimal standard curves were applied to individual assays and the absorbance values were detected at 450 nm using a microplate reader.

### qPCR and RT–PCR analyses

A QIAzol-based protocol was used for the extraction of RNAs from tissue samples. Briefly, a QIAzol lysis reagent (Cat. No. 79306, Qiagen) was added to small pieces of tissues followed by homogenization. The samples were incubated on ice for 15 min before adding chloroform and thoroughly mixed by vortex followed by 20 min centrifugation. RNA-containing supernatants were mixed with 100% ethanol and subsequently applied to a RNA extraction column provided in the RNA extraction kit (Cat. No. K0732, Thermo Scientific). All the procedures were performed according to the manufacturer's instruction. For RNA extraction from cells, a 2-mercaptoethanol (Cat. No. 3148, Sigma)-containing buffer was applied to lysed cells. Total RNA concentrations were measured with a nanodrop (Thermo Scientific) and an equal amount of RNA from each sample was used for cDNA synthesis using a RevertAid cDNA synthesis kit (Cat. No. K1632, Thermo Scientific). The cDNAs were subsequently used for qPCR with SYBR Green (Cat. No. 4367659, Life Technologies) using a StepOnePlus detectable system (Applied Biosystems); or the RT–PCR analysis with DreamTaq (Thermo Scientific) amplification in a thermal cycler (Cat. No. 2720, Applied Biosystems). The amplified products were separated on a GelRed (Cat. No. 41003, Biotium)-supplemented agarose gels and were detected in a Gel Doc XR^+^ (Bio-Rad). The reactions and PCR cycles were performed according to standard protocols recommended for SYBR Green and DreamTaq. All the qPCR data were presented as relative quantification and *Gapdh* was used as an internal control. Beta actin was used as loading control for RT–PCR analysis. The primers used in our experiments were shown as follows: m*Gapdh* forward: 5′-CCAGCAAGGACACTGAGCAA-3′, m*Gapdh* reverse: 5′-GGGATGGAAATTGTGAGGGA-3′ m*Il33* forward: 5′-ATGGGAAGAAGCTCATGCTG-3′, m*Il33* reverse: 5′-CCGACGACTTTTTCTGAAGG-3′; m*Sox7* forward: 5′-GACACCTTGGATCAGCTAAGCC-3′, m*Sox7* reverse: 5′-CCTCCAGCTCTATGACACACTG-3′; m*St2* forward: 5′-ATTCAGGGGACCATCAAGTG-3′, m*St2* reverse: 5′-CGTCTTGGAGGCTCTTTCTG-3′; mβ*actin* forward:5′-AGGCCCAGAGCAAGAGAGG-3′, mβ*actin* reverse:5′-TACATGGCTGGGGTGTTGAA-3′, h*Pdgfrb* forward: 5′-GAGATGCTGAGTGACCACTC-3′, h*Pdgfrb* reverse: 5′-CGAATGGTCACCCGAGTTTG-3′; h*Gapdh* forward: 5′-CATTTCCTGGTATGACAACGA-3′, h*Gapdh* reverse: 5′-GTCTACATGGCAACTGTGAG-3′.

### Immunohistochemistry

The PFA-fixed tumour tissues were embedded in paraffin, cut to 5-μm-thick sections using a microtome, and transferred onto glass slides. After baking, the slides were processed through serial steps to deparaffinize in Tissue-Clear (Cat. No. 1466, Sakura) and rehydrate tissues in 99–95–70% ethanol. For cryosection staining, tissue slides were fixed with acetone (Cat. No. 32201, Sigma). Rehydrated tissues were washed in PBS, boiled in a unmasking solution and subsequently blocked with 4% serum before incubation overnight at 4 °C with primary antibodies against F4/80 (Rabbit, Cat. No. NBP2-12506, Novus Biologicals), Iba1 (rabbit, Cat. No. 019-19741, WAKO), PDGFRα (Rat, Cat. No. 14-1401-82, eBioscience), PDGFRβ (Rat, Cat. No. 14-1402-81, eBioscience), NG2 (Rabbit, Cat. No. AB5320, Millipore) or αSMA (mouse, Cat. No. M0851, DAKO). The tissue slides were incubated for 30 min with fluorescent-labelled secondary antibodies including: an Alexa Fluor 555-labelled goat anti-rat antibody (Cat. No. A21434, Invitrogen); an Alexa Fluor 488-labelled donkey anti-mouse (Cat. No. A21202, Invitrogen); and an Alexa Fluor 488-labelled donkey anti-rabbit (Cat. No. A21206, Invitrogen) antibody. The slides were thoroughly washed and mounted with Vectashield containing DAPI. The positive signals were analysed under a fluorescence microscope (Zeiss LSM510 Confocal, or Nikon C1 Confocal microscope).

### H&E staining

The paraffin-embedded tumour and healthy tissues were cut into 5-μm-thick sections and mounted onto glass slides, which were deparaffinized in Tissue-Clear (Cat. No. 1466, Sakura) and rehydrated in 99–95–70% ethanol. The tissue slides were stained with haematoxylin and counterstained with eosin before being dehydrated with 95–99% ethanol and mounted with PERTEX (Cat. No. 00801, HistoLab). The stained tissues were analysed under a light microscope (Nikon Eclipse TS100).

### FACS analysis

At the tumour size of approximately 1.5 cm^3^, fresh blood was intracardiacally collected using a heparinized syringe immediately after the mice were killed. About 2.5 ml of a red blood cell lysis buffer (Cat. No. 00-4333-57, eBioscience) was added to 250 μl of fresh blood from each mouse. After 3 min incubation, the lysis reaction was stopped by the addition of 10 ml PBS. After centrifugation, the cell pellets were resuspended in 1% PFA–PBS. The cell samples were analysed by FACS (BD). Healthy mouse blood and RFP^+^ tumour cells were used as controls. For TAMs analysis, freshly dissected subcutaneous tumour tissues were collected, followed by immediately cutting into small pieces and digested with 0.15% collagenases I and II at 37 °C for 60 min. The single-cell suspension was prepared by a 0.40-μm filter. The cells were stained for 45 min on ice with primary antibodies. An Alexa Fluor 647 anti-mouse F4/80 antibody (Cat. No. 123122, BioLegend), a PE anti-mouse CD206 antibody (Cat. No. 141705, BioLegend), a FITC-labelled anti-mouse CCR3 antibody (Cat. No. 144509, BioLegend), an anti-mouse PDL2 antibody (Cat. No. 107202, BioLegend) and an anti-mouse ST2 antibody (Cat. No. 145301, BioLegend) were used as primary antibodies. An Alexa Fluor 555 anti-rat antibody (Cat. No. A21434, Invitrogen) and an Alexa Fluor 488 anti-rat antibody (Cat. No. A21208, Invitrogen) were used as secondary antibodies. For Ki67 and CTC analysis, before primary antibody staining, the cells were permeabilized with 0.3% Triton X-100 in PBS for 10 min. The stained cells were analysed by FACScan (BD) or MoFlo XTD (Beckman Coulter).

### Adenovirus

An adenovirus-GFP (Adv-*Gfp*, 1 × 10^9^ PFU) and an adenovirus vector expressing PDGF-BB (Adv-*Pdgfb*, 1 × 10^8^ PFU) were intravenously injected into each of the immunodeficient SCID mice on day 0 or day 3. The mice were killed at day 14 after virus injection. The mouse livers were used for immunohistochemistry and qPCR analyses.

### Statistical analysis

The sample sizes were carefully chosen for each experiment on the basis of pilot experiment examinations and sufficient statistic powers. For all animal studies, at least eight animals per group were used to ensure the adequate power. The animals were excluded from the analysis if they did not meet the pre-established criteria of the Karolinska Institute Template. In all animal experiments, the experimental animals were randomly and blindly divided into each group to receive various treatments. A standard two-tailed Student's *t*-test was used for all the statistical analyses. All the sample sizes were appropriate for assumption of normal distribution and variance was similar between the compared groups. The statistical values of *P*<0.05, *P*<0.01 and *P*<0.001 were considered statistically significant. The values of mean determinants are presented as ±s.e.m.

## Additional information

**Accession codes:** Gene-array data were deposited in the Gene Expression Omnibus with accession numbers GSE46564 and GSE69402.

**How to cite this article:** Yang, Y. *et al*. The PDGF-BB-SOX7 axis-modulated IL-33 in pericytes and stromal cells promotes metastasis through tumour-associated macrophages. *Nat. Commun.* 7:11385 doi: 10.1038/ncomms11385 (2016).

## Supplementary Material

Supplementary Information Supplementary Figures 1-10
