# Supplementary material for: The PDGF-BB-SOX7 axis-modulated IL-33 in pericytes and stromal cells promotes metastasis through tumour-associated macrophages
Source: Nat Commun. 2016 May 6;7:11385. doi: 10.1038/ncomms11385 (PMC4859070; doi:10.1038/ncomms11385)
Supplement: Supplementary Information — Supplementary Figures 1-10 [file ncomms11385-s1.pdf]

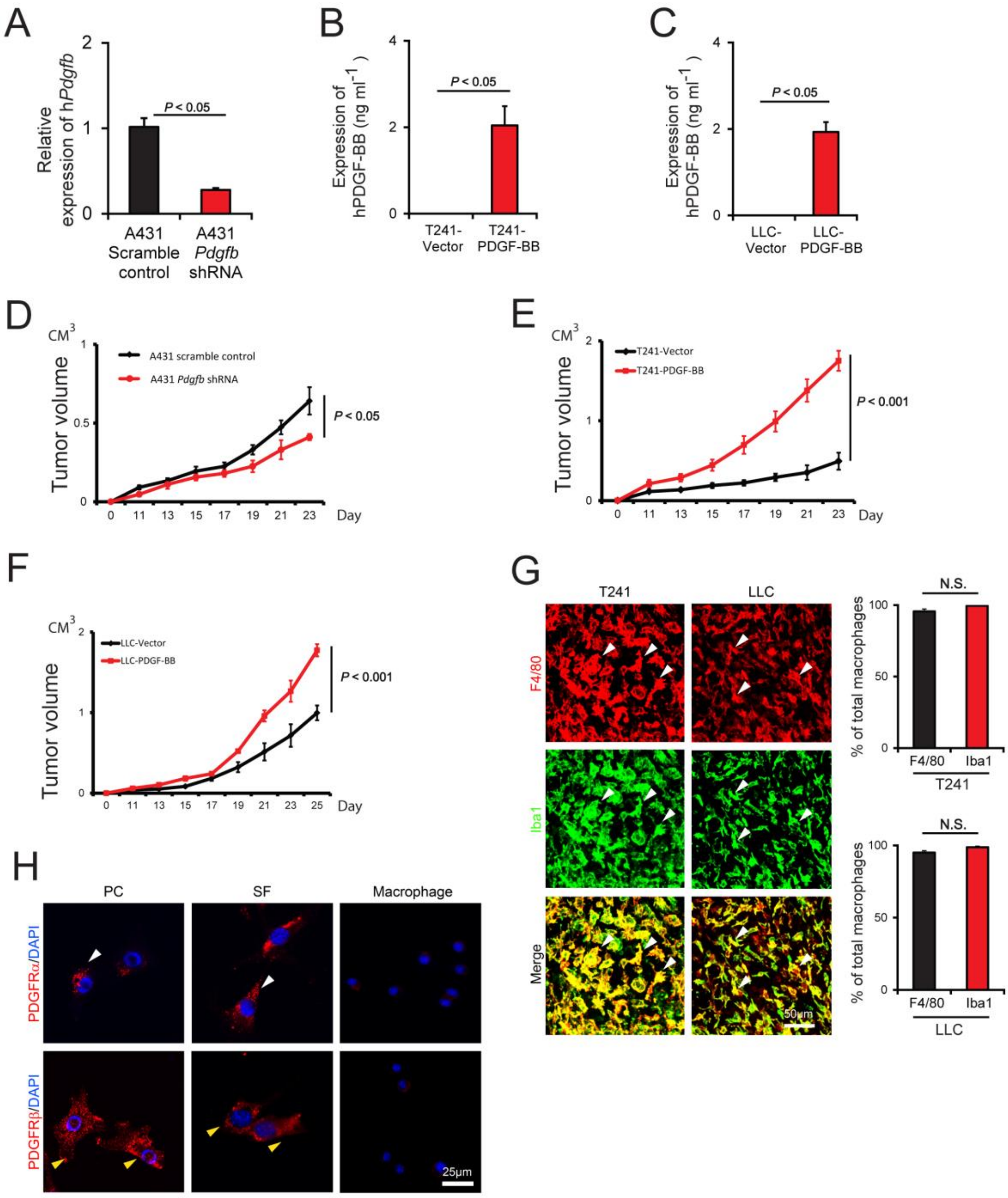

Supplementary Fig. 1. Gain-of-function and loss-of-function of PDGF-BB in tumor growth

a) Analysis of *Pdgfb* mRNA expression by qPCR in scramble- and *Pdgfb*-shRNA-transfected A431 tumor cells. (n = 6 samples/group).

b) PDGF-BB protein levels in vector- and PDGF-BB-transfected T241 tumor cells. (n = 6 samples/group).

c) PDGF-BB protein levels in vector- and PDGF-BB-transfected LLC tumor cells. (n = 6 samples/group).

d) Tumor growth curve of scramble- and *Pdgfb*-shRNA-transduced human A431 squamous carcinoma xenograft. (n = 8 mice/group).

e) Tumor growth curve of vector- and PDGF-BB-transfected T241 fibrosarcomas xenograft. (n = 8 mice/group).

f) Tumor growth curve of vector- and PDGF-BB-transfected LLC carcinomas xenograft. (n = 8 mice/group).

g) Double immunostaining of $Iba1^+$ signals (green) and $F4/80^+$ signals (red) in T241 and LLC tumor tissues. Arrowheads point to $F4/80^+$-$Iba1^+$ macrophages. Quantifications of $Iba1^+$ and $F4/80^+$ macrophages in T241 (n = 8 random fields/group) and LLC tumor tissues. (n = 8 random fields/group).

h) Immunostaining of PDGFRα and PDGRβ in pericytes, stromal fibroblasts and macrophages. White arrowheads indicate PDGFRα positive signals. Yellow arrowheads indicate PDGFRβ positive signals. Bar = 25 μm. PC = pericytes, SF = stromal fibroblasts (mean ± s.e.m., N.S. = not significant, Student's t-test).

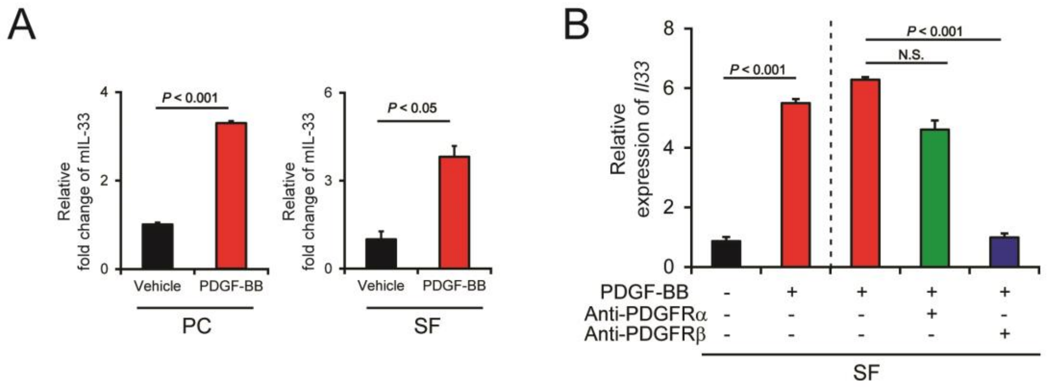

Supplementary Fig. 2. PDGF-BB-PDGFRβ-signalling induces IL-33 expression in pericytes and stromal fibroblasts

a) IL-33 protein levels in vehicle- or PDGF-BB-treated lung pericytes and bone marrow stromal fibroblasts cultured in vitro (n = 6 samples/group). PC = pericytes. SF = stromal fibroblasts.

b) qPCR analysis of *Il33* mRNA of vehicle-, anti-PDGFRα-, or anti-PDGFRβ-treated PDGF-BB-stimulated or non-stimulated stromal fibroblasts (SF; n = 6 samples/group). N.S. = not significant (mean ± s.e.m., N.S. = not significant, Student’s t-test).

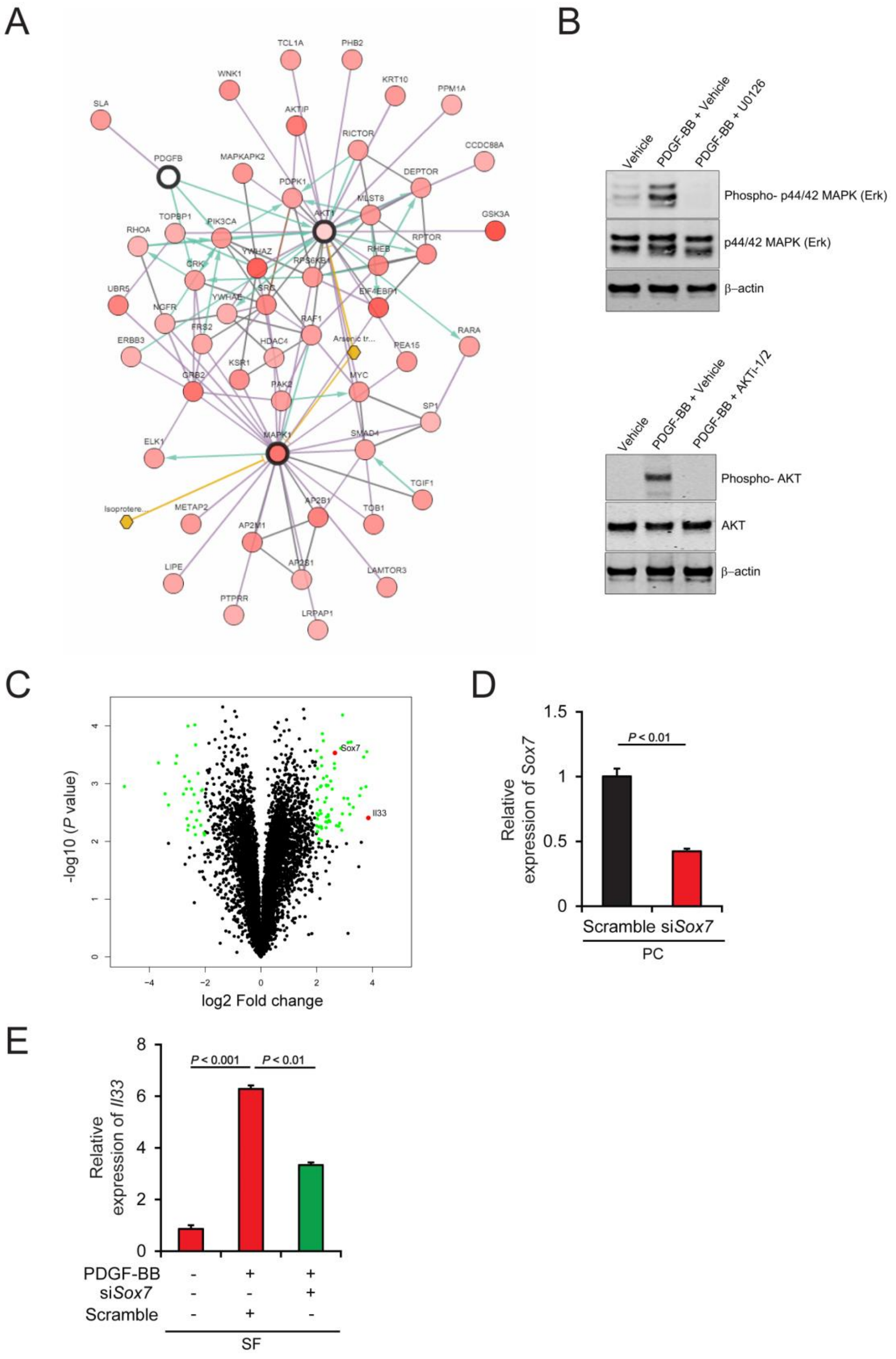

Supplementary Fig. 3. SOX7 transcription factor mediates PDGF-BB- induced IL-33 expression

a) Network analysis of expression relation between *Pdgfb, Akt1* and *Mapk1* expression in clinical human patients. Multiple datasets were applied to analyses. *Pdgfb*, *Akt1* and *Mapk1* were highlighted.

b) Immunoblot detection of total and phosphorylated MAPK (Erk), total and phosphorylated Akt in vehicle-, U0126-, and Akti-1/2-treated PDGF-BB-stimulated pericytes at 10 min. Vehicle-treated PDGF-BB-non-stimulated pericytes were used as controls.

c) Volcano plot comparing genome-wide gene expression of PDGF-BB-stimulated- and non-stimulated- pericytes. Genes with fold change > 4 and P < 0.01 were highlighted in green dots. *Sox7* and *Il33* were highlighted in red dots.

d) qPCR quantification of *Sox7* mRNA expression levels in scramble- or *Sox7*-siRNA-treated lung pericytes (PC; n = 6 samples/group).

e) qPCR analysis of *Il33* mRNA expression levels of vehicle- or PDGF-BB-stimulated stromal fibroblasts that were transfected with scrambled or *Sox7*-siRNA (n = 6 samples/group). SF = stromal fibroblasts (mean ± s.e.m., Student's t-test). Full-gel images for panel b are shown in Supplementary Fig. 10.

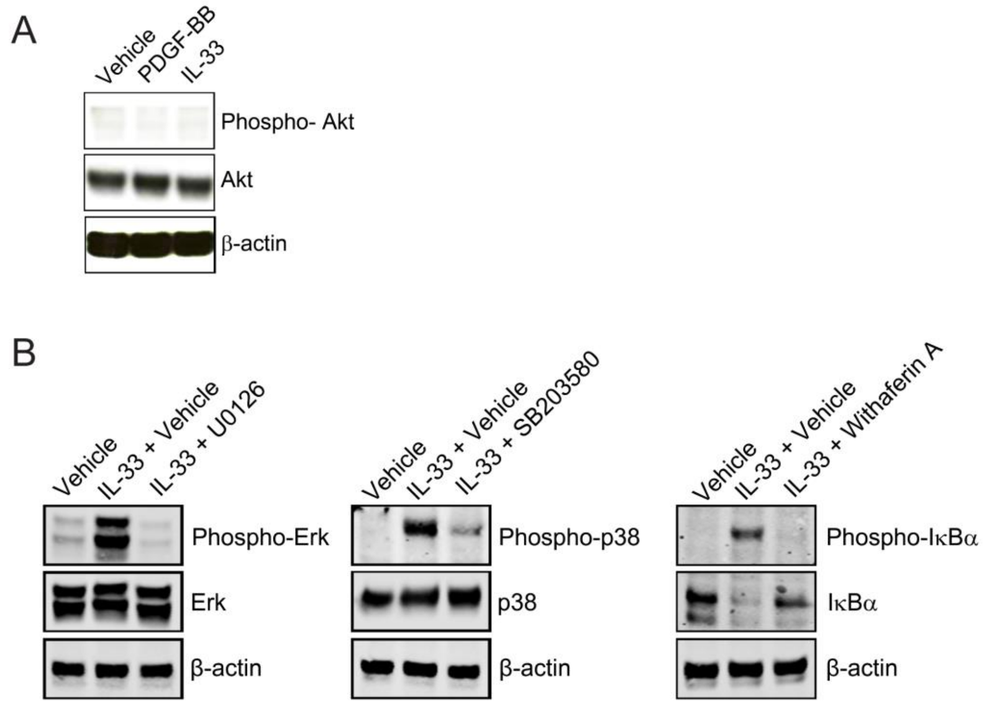

Supplementary Fig. 4. Validation of signalling inhibitors in IL-33-stimulated macrophages

a) Total and phosphorylated Akt in vehicle-, PDGF-BB- and IL-33-stimulated macrophages at 10 min. Beta-actin indicates the loading level in each lane.

b) Immunoblot detection of total and phosphorylated MAPK (Erk), p38, and IκBα in vehicle- and their specific inhibitor-treated IL-33-stimulated macrophages. Vehicle-treated IL-33-non-stimulated macrophages were used as controls. Full-gel images for panels a-b are shown in Supplementary Fig. 10.

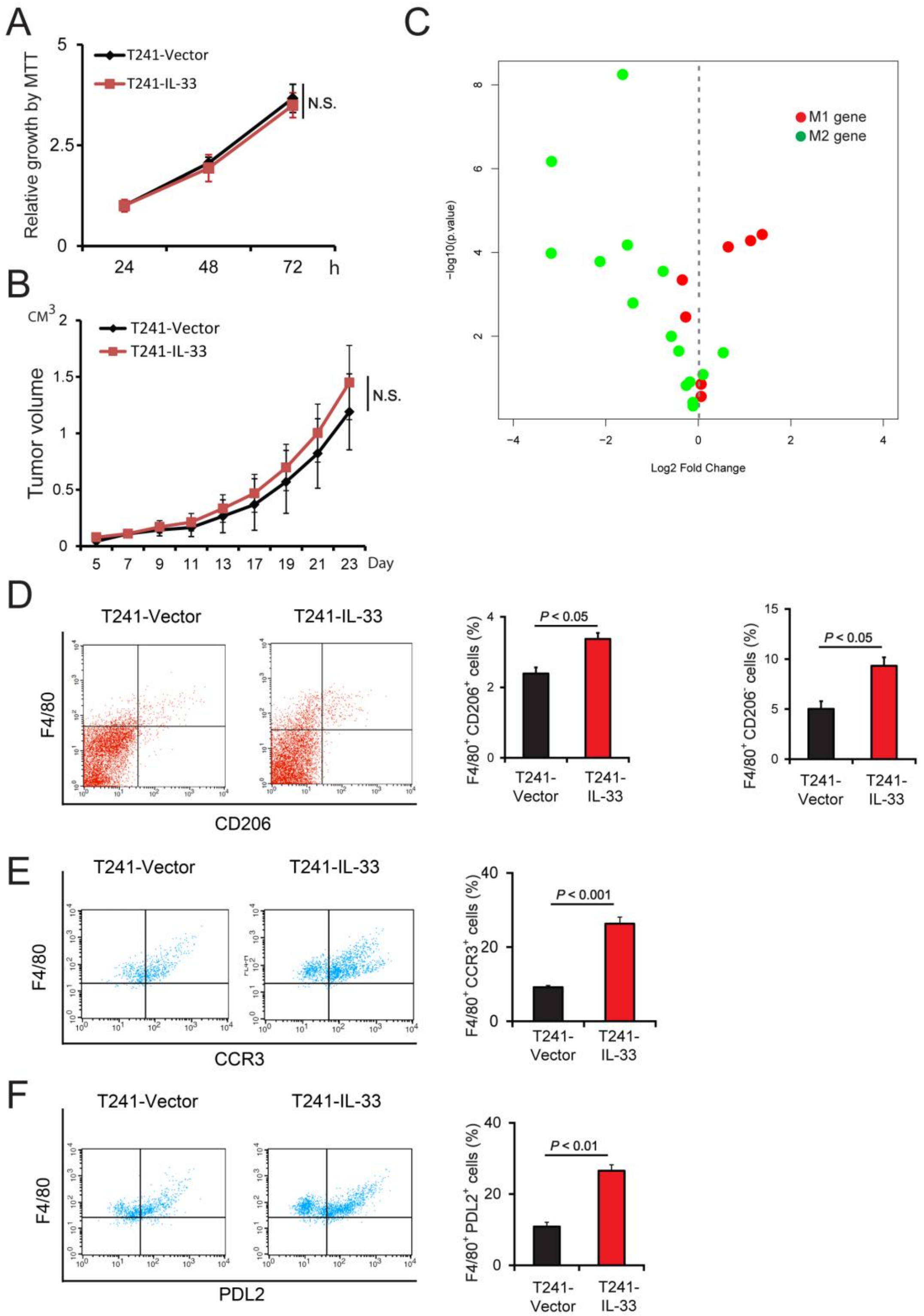

Supplementary Fig. 5. Primary tumor growth and macrophage phenotypes of IL-33-expressing and vector T241 tumors

a) *In vitro* tumor cell proliferation rates of vector- or IL-33-T241 cells. (n = 6 samples/group).

b) *In vivo* tumor growth rates of vector- or IL-33-T241 fibrosacoma xenograft. (n = 8 mice/group).

c) Volcano plot of M1 (red) and M2 (green) related genes by genome-wide expression profiling of F4/80$^+$ cells isolated from tumors implanted in *wt* and *St2-/-* mice (n = 3 samples/group).

d) FACS analysis of the F4/80$^+$CD206$^+$ macrophages in vector- and IL-33-T241 tumor tissues (n = 5 samples/group). Quantification of percentages of F4/80$^+$CD206$^+$ and F4/80$^+$CD206$^-$ macrophage subpopulations.

e) FACS analysis of the F4/80$^+$CCR3$^+$ macrophages in vector- and IL-33-T241 tumor tissues (n = 5 samples/group). Quantification of percentages of F4/80$^+$CCR3$^+$ subpopulations in F4/80$^+$ macrophage populations.

f) FACS analysis of the F4/80$^+$PDL2$^+$ macrophages in vector- and IL-33-T241 tumor tissues (n = 5 samples/group). Quantification of percentages of F4/80$^+$PDL2$^+$ subpopulations in F4/80$^+$ macrophage populations (mean ± s.e.m., N.S. = not significant, Student's t-test).

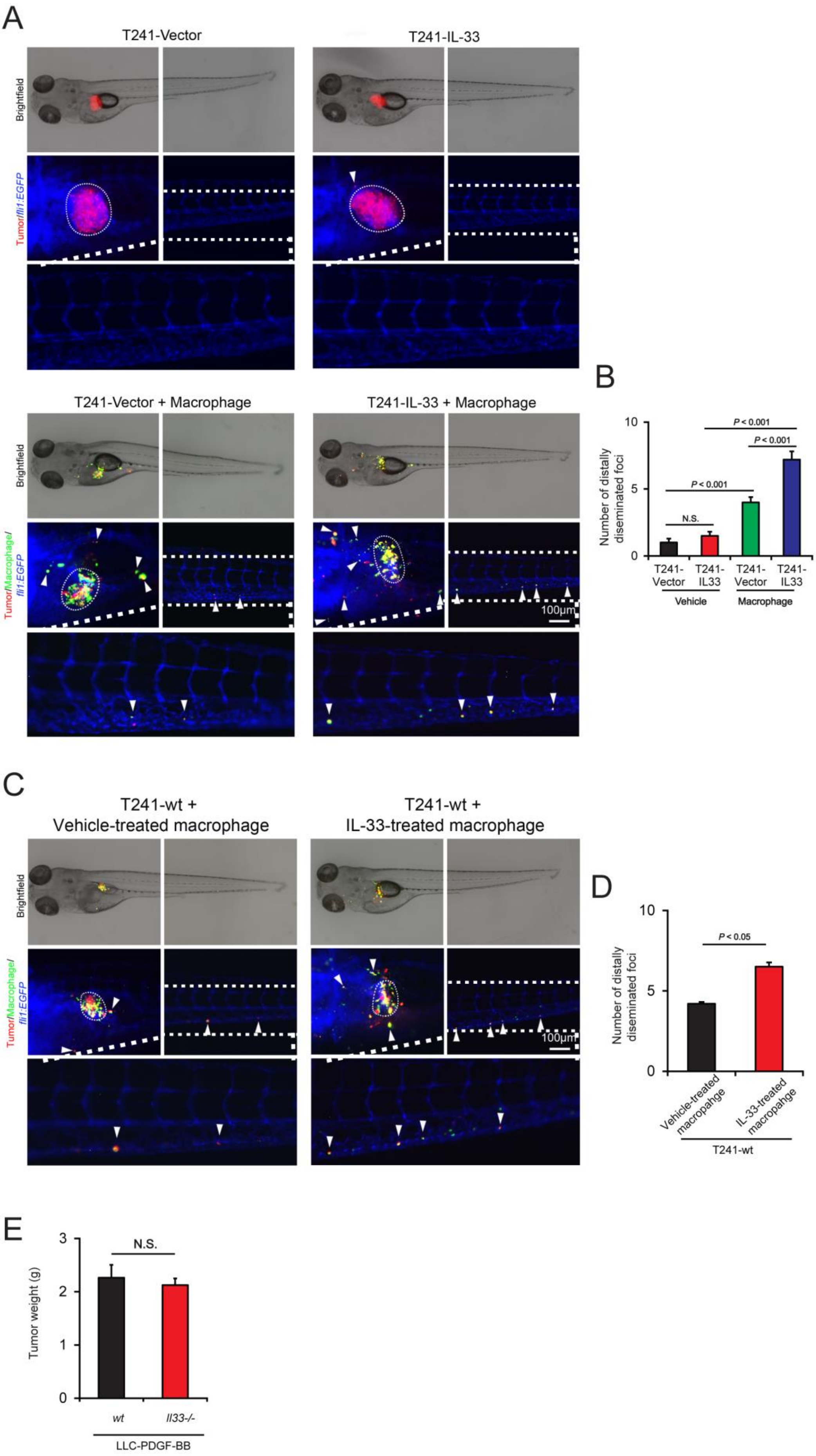
A
T241-Vector
T241-IL-33
Brightfield
Tumor/fli1:EGFP
T241-Vector + Macrophage
T241-IL-33 + Macrophage
Tumor/Macrophage/fli1:EGFP
100μm
B
Number of distally diseminated foci
10
5
0
P < 0.001
P < 0.001
P < 0.001
N.S.
T241-Vector
T241-IL33
T241-Vector
T241-IL33
Vehicle
Macrophage
C
T241-wt + Vehicle-treated macrophage
T241-wt + IL-33-treated macrophage
D
P < 0.05
Vehicle-treated macropahge
IL-33-treated macropahge
T241-wt
E
Tumor weight (g)
3
2
1
0
N.S.
wt
Il33-/-
LLC-PDGF-BB

Supplementary Fig. 6. IL-33-stimulated macrophages promotes metastasis in zebrafish

a) RFP$^+$ vector- and IL-33-T241 tumor metastasis in zebrafish. Tumor cells either alone or together with macrophages (green) were implanted into the PVS of each zebrafish embryo and metastasis were monitored at day after implantation. Dashed lines encircle primary tumors. Arrowheads indicate proximal and distal metastases. Bar = 100 μm.

b) Quantification of distal metastases in zebrafish embryos (n = 20 embryos/group). N.S. = not significant.

c) Micrographs of zebrafish embryos implanted with T241 tumor cells (red) together with vehicle- or IL-33-treated macrophages (green). Dashed lines encircle primary tumors. Arrowheads indicate proximal and distal metastases. Bar = 100 μm.

d) Quantification of distal metastases in zebrafish embryos (n = 20 embryos/group).

e) Tumor weight of PDGF-BB-LLC tumors implanted in *wt* and *Il33-/-* mice (n = 6 animals/group) (mean ± s.e.m., N.S. = not significant, Student's t-test).

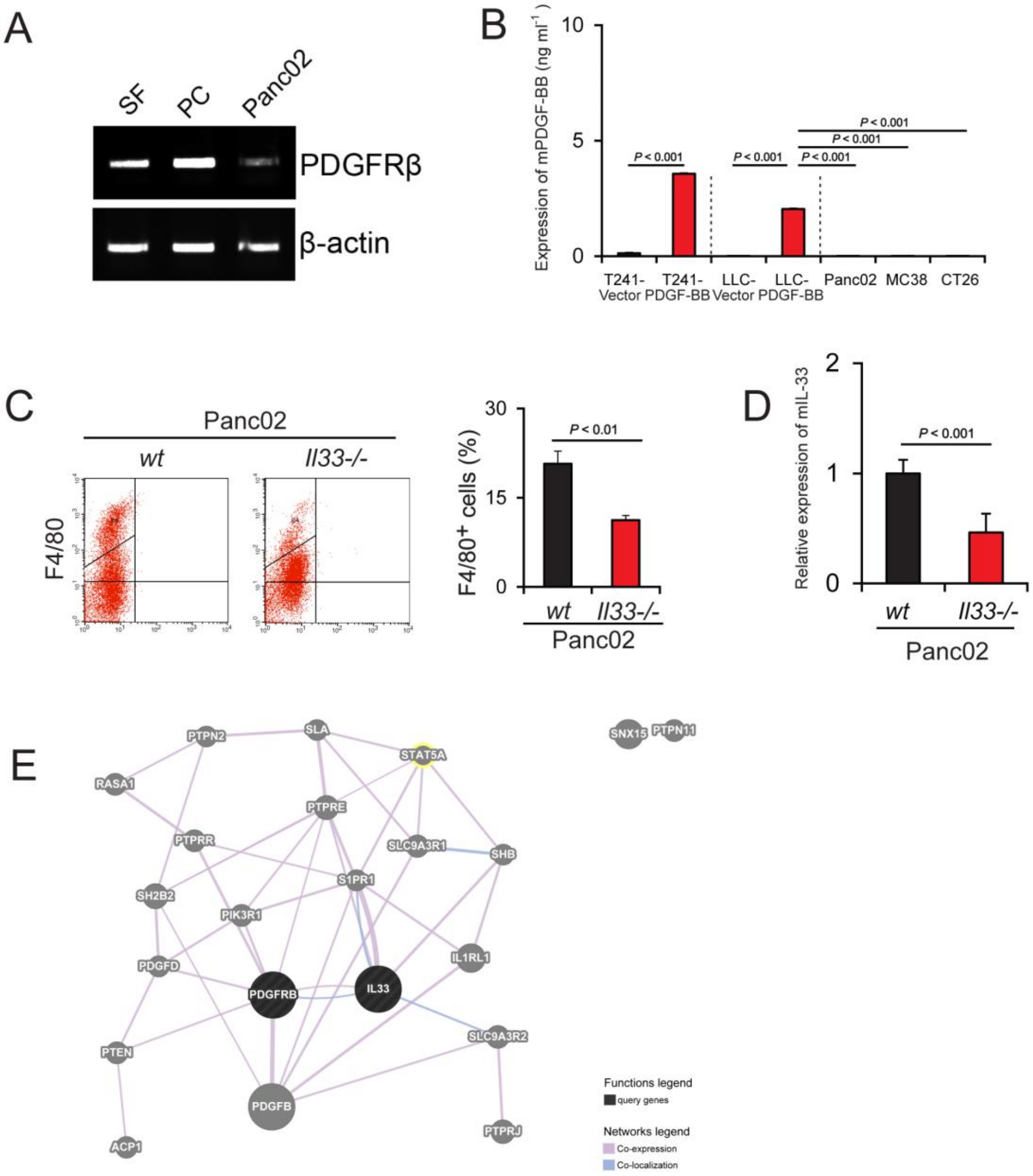

Supplementary Fig. 7. Relation of stromal PDGFRβ to IL-33 expression pancreatic tumors

a) RT-PCR analysis of *Pdgfrb* mRNA levels in stromal fibroblasts, pericytes, and Panc02 tumor cells. Beta-actin was used as a standard loading.

b) ELISA detection of PDGF-BB protein expression levels in various cultured tumor cell lines (n = 6 samples/group). PDGF-BB-T241 and PDGF-BB-LLC served as positive controls.

c) FACS analysis of the F4/80$^+$ macrophages in Panc02 tumor implanted in *wt* and *Il33-/-* mice (n = 5 samples/group). Quantification of percentages of F4/80$^+$ macrophage subpopulations.

d) ELISA detection of IL-33 protein expression levels in tumor tissues of Panc02-tumor-bearing *wt* and *Il33-/-* mice. (n = 6 samples/group).

e) Network analysis of expression relation between *Pdgfrb* and *Il33* expression in clinical human patients. Multiple datasets were applied to analyses. *Pdgfrb* and *Il33* were highlighted in black colour (mean ± s.e.m., Student's t-test).

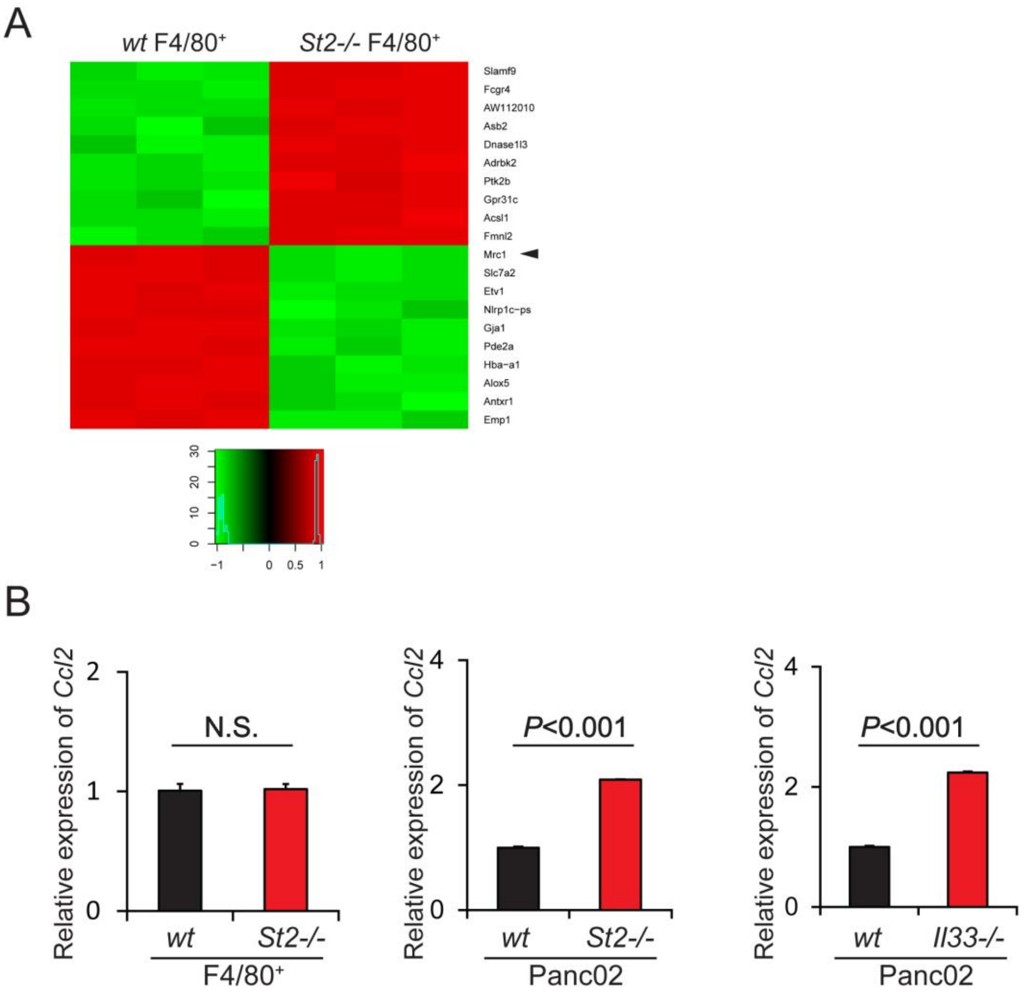

Supplementary Fig. 8. Genome-wide expression profiling of *wt* and *St2-/-*TAMs

a) Heatmap of top 10 up and down differentially regulated genes by genome-wide expression profiling of F4/80$^+$ cells isolated from Panc02 tumors implanted in *wt* and *St2-/-* mice (n = 3 samples/group).

b) qPCR analysis of *Ccl2* mRNA expression of F4/80$^+$ cells isolated from tumors implanted in *wt* and *St2-/-* mice (n = 6 samples/group). qPCR analysis of *Ccl2* mRNA expression of whole Panc02 tumor tissues implanted in *wt*, *St2-/-*, and *Il33-/-* mice (n = 6 samples/group) (mean ± s.e.m., N.S. = not significant, Student's t-test).

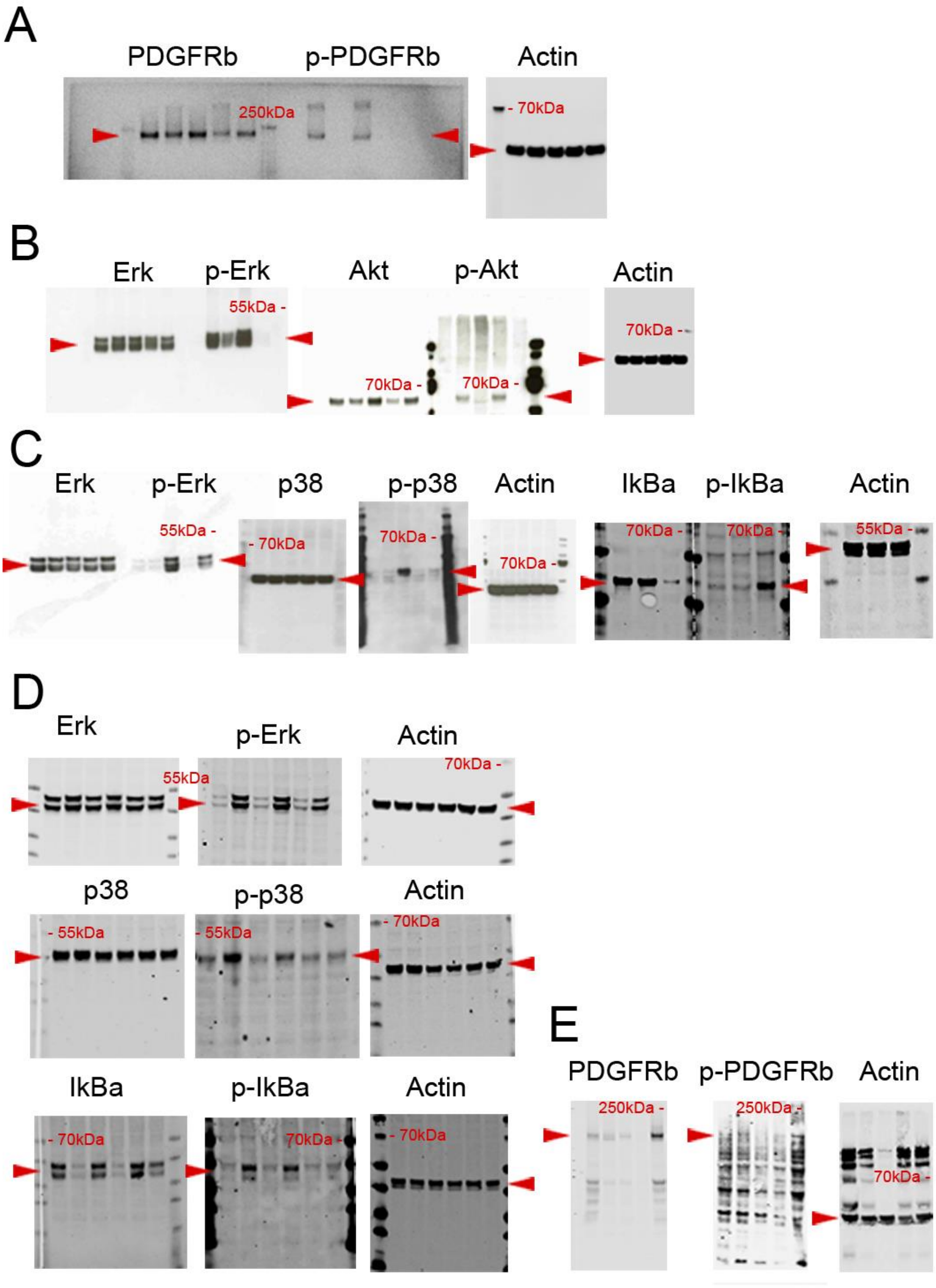

Supplementary Fig. 9. Original full-length western blots used for Figures 3,4, and 7

a) Original western blots used for Fig. 3a.

b) Original western blots used for Fig. 3b.

c) Original western blots used for Fig. 4g.

d) Original western blots used for Fig. 4h.

e) Original western blots used for Fig. 7c.

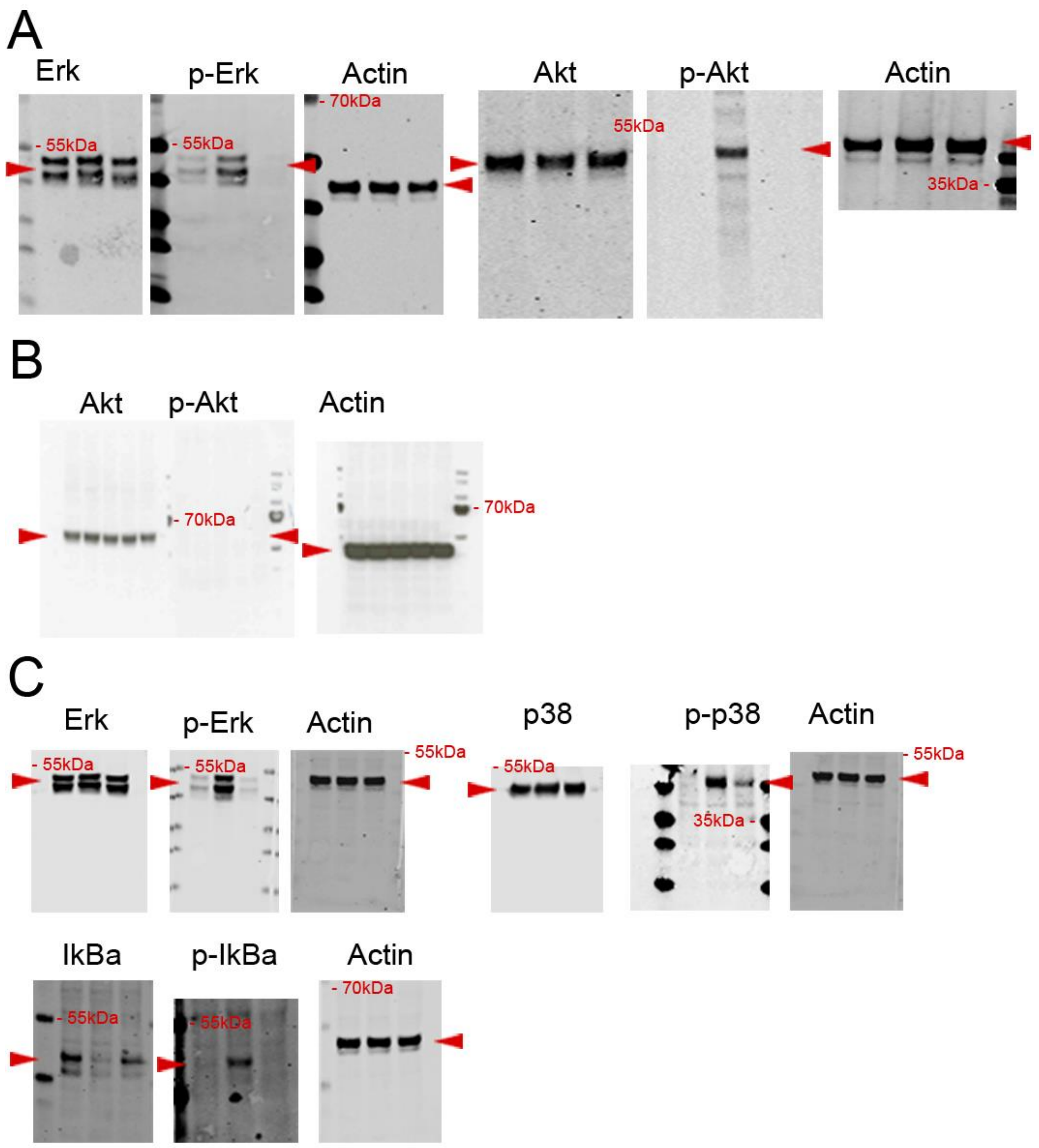

Supplementary Fig. 10. Original full-length western blots used for Supplementary Figures 3 and 4

a) Original western blots used for Supplementary Fig. 3b.

b) Original western blots used for Supplementary Fig. 4a.

c) Original western blots used for Supplementary Fig. 4b.
